# The Biological Fate of Silver Nanoparticles from a Methodological Perspective

**DOI:** 10.3390/ma11060957

**Published:** 2018-06-05

**Authors:** Damjana Drobne, Sara Novak, Iva Talaber, Iseult Lynch, Anita Jemec Kokalj

**Affiliations:** 1 Department of Biology, Biotechnical Faculty, University of Ljubljana, Ljubljana 1000, Slovenia; damjana.drobne@bf.uni-lj.si (D.D.); sara.novak@bf.uni-lj.si (S.N.); anita.jemec@bf.uni-lj.si (A.J.K.); 2 School of Geography, Earth and Environmental Sciences, University of Birmingham, Birmingham B15 2TT, UK; I.Lynch@bham.ac.uk

**Keywords:** silver nanoparticles review, body burden, biodistribution, internalization, bioaccumulation, microscopy, spectroscopy, throughput, performance, quantification

## Abstract

We analyzed the performance and throughput of currently available analytical techniques for quantifying body burden and cell internalization/distribution of silver nanoparticles (Ag NPs). Our review of Ag NP biological fate data shows that most of the evidence gathered for Ag NPs body burden actually points to total Ag and not only Ag NPs. On the other hand, Ag NPs were found inside the cells and tissues of some organisms, but comprehensive explanation of the mechanism(s) of NP entry and/or in situ formation is usually lacking. In many cases, the methods used to detect NPs inside the cells could not discriminate between ions and particles. There is currently no single technique that would discriminate between the metals species, and at the same time enable localization and quantification of NPs down to the cellular level. This paper serves as an orientation towards selection of the appropriate method for studying the fate of Ag NPs in line with their properties and the specific question to be addressed in the study. Guidance is given for method selection for quantification of NP uptake, biodistribution, precise tissue and cell localization, bioaccumulation, food chain transfer and modeling studies regarding the optimum combination of methods and key factors to consider.

## 1. Introduction and Problem Description

Silver nanoparticles (Ag NPs) are within the top five most studied nanomaterials in terms of their (eco)toxicological properties [1,2]. NP biological fate has emerged as a key aspect of toxicokinetics. Therefore, numerous studies have addressed the whole organism body burden, organism distribution, cell/tissue internalization, and trophic transfer of NPs [3,4]. The study of the biological fate of Ag NPs is very challenging because these NPs undergo a number of transformations in the test medium during exposure as well as in the organism following uptake and internalization. Under ambient conditions, Ag NPs undergo slow oxidation, and, as a result, ionic silver (Ag^+^) is released [5,6]. The chemical reactions responsible for the dissolution of Ag NPs involve redox reactions in the presence of either oxygen or hydrogen peroxide [7]. The amount of dissolution depends on the intrinsic properties of the Ag NPs (particle size, surface modification (capping), shape, surface morphology), and the characteristics of the surrounding medium (pH, ionic strength, ionic composition) (e.g., Cl^−^ is known to enhance Ag NP dissolution [8] while sulfur can slow it through formation of a sulfide layer at the surface that inhibits further dissolution [9]) and the presence of organic molecules (e.g., proteins, polysaccharides, and natural organic matter) [6,10,11]. Ag NPs have also been shown to undergo slow ageing during storage. Therefore, an initial assessment of the concentration of ionic Ag^+^ in Ag NP test suspensions needs to be undertaken before and during exposure to the biological system [12]. Furthermore, it has been shown that Ag NPs may form secondary NPs inside the organism by precipitation of intracellular Ag^+^. For example, this phenomenon was found in the shoots of wheat (*Triticum aestivum*) [13], in *Arabidopsis thaliana* [14], in the leaf tissue of lettuce (*Lactuca sativa*) [15], *Brassica juncea* (Indian mustard), *Medicago sativa* (Alfalfa), and *Helianthus annuus* (Sunflower) [16] exposed only to AgNO_3_ and not Ag NPs. Additionally, in the vacuoles of *Tetrahymena thermophila*, Ag NPs were formed from Ag^+^ [17]. The observation of in vivo biosynthesis of Ag NPs is not surprising because various types of microorganisms have also been shown to synthesize Ag NPs [18,19]. Ag NP biosynthesis is one of the possible detoxification mechanisms where insoluble composites are formed, reducing the bioavailability of Ag [17]. However, this phenomenon can confound the interpretation of external Ag NP uptake by the organism, because the detected NPs inside the organism could actually be secondary NPs, formed by the precipitation of internalized Ag^+^ ions, originating from the dissolution of the initial Ag NPs or from residuals from Ag NP synthesis process.

Our primary intention in this study was to review the literature on the biological fate of Ag NPs in organisms commonly used in environmental studies, with the aim of providing definitive evidence-based, method-appropriate conclusions regarding the body burden and internalization/distribution of Ag NPs in organisms (Tables S1 and S2). However, the review showed that no definite conclusion regarding Ag NPs biological fate can be provided: in some studies, evident distribution of Ag NPs within different body regions was reported using appropriate methodologies with sufficient detection capabilities, but in other studies testing the same species, Ag NPs internalization was not recorded (Table S2). As an example, a number of studies have been done with the nematode *Caenorhabditis elegans* and various types of Ag NPs, but contradicting conclusions were reported. Kim et al. [20] found no internalization of citrate-coated 50.6-nm (primary particle size) Ag NPs after a 24 h exposure of *C. elegans* to 100 mg/L Ag NPs in nematode agar growth medium, whereas Roh et al. [21] found 20-nm non-coated Ag NPs around the uterine area after 24 h exposure to 0.5 mg/L in the same medium. Evidence of Ag NPs distribution throughout *C. elegans* was found after 24 h of exposure to 54 mg/L citrate-coated 7-nm Ag NPs dispersed in K^+^ liquid medium [22]. Yang et al. [23] found citrate-coated (25 nm, 10 mg Ag/L) Ag NPs dispersed in reconstituted hard water primarily in the gut of *C. elegans*, but they report different results for other tissues depending on the method (TEM coupled with EDX or hyperspectral imaging) used to detect the NPs.

There are a number of factors that could explain the observed variation in Ag NPs internalization/bioaccumulation data in various species, such as differences in Ag NPs properties (size, coating, and charge), different dispersion protocols, widely differing NP concentrations which can result in very different degrees of agglomeration and agglomerate sizes, exposure durations, test media composition and pH and different test organisms [24]. However, we were unable to pinpoint a single factor that could explain the observed variability in Ag NPs biological fate (Table S2). Most probably a combination of Ag NPs properties together with biomolecular corona jointly contributes to the biological identity of NPs which governs their distribution and toxicity [25]. We propose that the contradicting results regarding the biological fate of Ag NPs may also arise from inadequate understanding of the complex characteristics of Ag NPs transformations and of the performance and limitations of the methods applied in the studies. In particular, the distinction between internalized NPs and ions and potentially between NPs taken up intact versus NPs precipitated from internalized ions needs consideration. In recent years, a number of studies have already reviewed analytical approaches to support detection, quantification, and characterization of NPs [2,26,27]. However, none of these studies specifically address the environmental fate of Ag NPs and critically evaluate the suitability of the methodology employed by studies in the available literature. 

An additional inconsistency in existing literature is the terminology used to address the bioaccumulation of NPs. A traditional definition of bioaccumulation is that the chemical is taken up and accumulated in higher concentrations than in its immediate environment [28]. This definition is also adopted by OECD (The Organisation for Economic Co-operation and Development) technical guidelines [29]. The main assumption here is that the chemical is passing epithelial barriers and is assimilated in the tissue. However in nanomaterial studies, authors commonly use the term NP bioaccumulation to refer to ingested material present in the gut, those adsorbed to the body surface, those entering from other body openings besides an oral route as well as those NPs precipitated internally by the organisms from accumulated ions (discussed further in Chapter 3) [30]. Hence, we propose the term “whole organism body burden” rather than “bioaccumulated NPs” when we refer to the total amount of NPs in the organism.

In this study, we compared and reviewed the performance and throughput of analytical techniques employed by different studies of body burden and internalization/distribution of Ag NPs published in the recent years, relying on our own experiences in the field on metal internalization, microscopy, and spectroscopy [31,32,33,34]. This provided the basis for construction of a decision scheme to support researchers in selecting the most suitable analytical methods according to their characteristics (advantages and limitations) and the nature of the scientific question to be addressed by the study. As an outcome, we provide guidance on the type of information derived from Ag NP biological fate studies depending on the method applied, as a means to improve the consistency and accuracy of the datasets emerging from these types of studies.

## 2. Analyses of Analytical Techniques for Quantification of NP Biological Fate in Terms of Their Characteristics and Throughput

**Figure 1:**
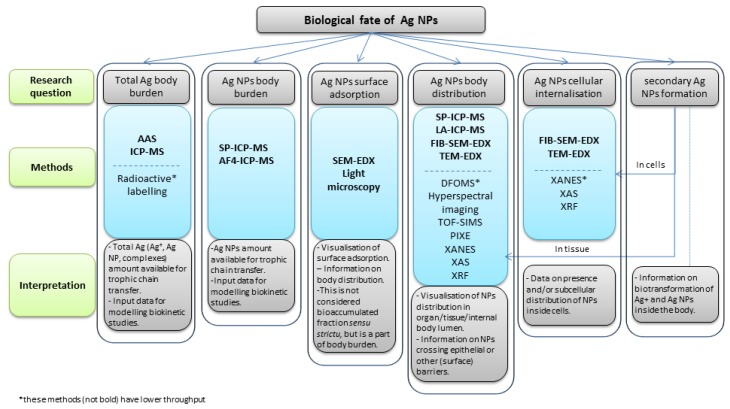
A decision scheme for selection of methods suitable for study of the biological fate of silver nanoparticles (Ag NPs) according to the specific research question.

There are a variety of techniques for studying the fate of NPs in biological samples [2,26,27]. However, there is no single technique that can discriminate between ions and NPs, and at the same time enable localization and quantification of NPs in biological samples [26]. When selecting a technique, a compromise should be made between the visualization and quantification ability and the discrimination between ions and particles as well as their throughput. To facilitate the choice of methods we reviewed the most widely used techniques for metal oxide NP biological fate investigation, focusing on their principle of detection, specificity, and limitations for measuring NPs in tissue (Supplementary Information, Chapter S2). Based on this extensive review, we extracted the most important information (Table 1) regarding their characterization capability and throughput, which served as the basis to design the decision scheme for selection of the most appropriate methods to study Ag NPs biological fate based on the specific question/endpoint of interest (Figure 1).

### 2.1. Characteristics of Techniques by Their Information Output

**Figure 2:**
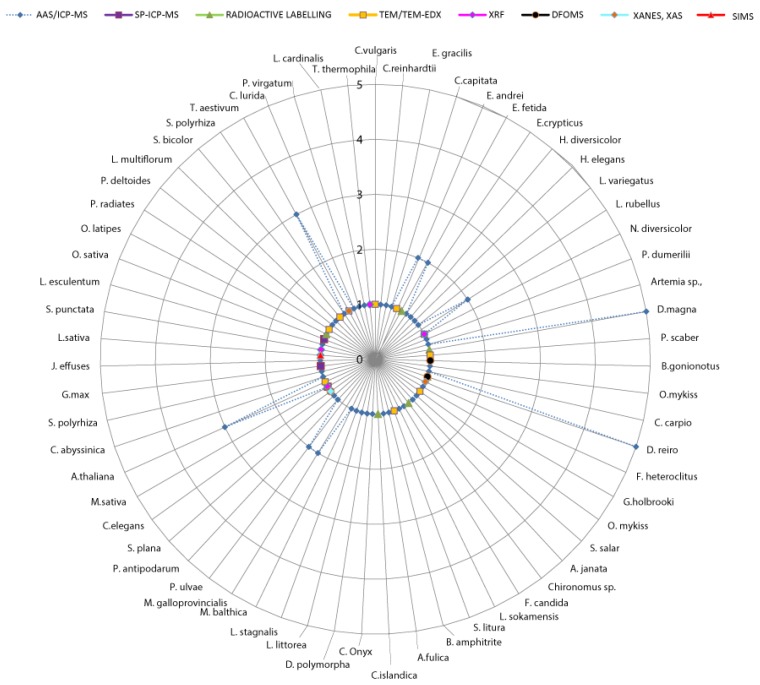
A diagram showing the distribution of Ag NPs studies for 63 different species using the techniques: AAS/ICP-MS, SP-ICP-MS; Radioactive labelling; TEM/TEM-EDX; XRF; DFOMS; XANES; XAS and SIMS. Organisms are grouped according to their taxonomic position. The number of studies is represented by radial scale numbered from 1–5 (each radius represents one study). Most of the studies were done on fish *Danio rerio* (total 8), crustacean *Daphnia magna* (6), and plants *Triticum aestivum* (6), Alfalfa *Medicago sativa* (4), *Arabidopsis thaliana* (4). The majority of species was tested only in one study.

In general, techniques could be grouped into those for quantification (of total metal and/or NPs) and those enabling visualization of NP cellular and tissue localization. The most frequently used methods for quantification of NPs inside the body of an organism are AAS and ICP-MS, which provide the total amount of metal species, while SP-ICP-MS and AF4-ICP-MS are able to distinguish NPs from ions (Figure 2) [35]. When sub-cellular localization is of interest, a compromise between the spatial resolution of the method and its throughput needs to be made. A good choice for intracellular localization of elements is TEM-EDX due to its high resolution [36], but internal cellular distribution could also be provided by FIB-SEM-EDX [20], as well as synchrotron-based techniques (XANES, XAS, XRF). Among the synchrotron-based techniques XANES is even able to discriminate between different elemental species [37] (Table 1A) and can thus potentially discriminate between intact NPs internalized from the external environment versus NPs precipitated in situ from internalized Ag^+^ ions. The emerging method of single cell SP-ICP-MS [38] may in the future provide a bridge between quantification and localization, and single cell LA-ICP-MS [39] is already making strides in this direction although its spatial resolution (~2.5 μm) is not sufficient to identify individual NPs.

In the case of NPs with high dissolution potential, such as Ag NPs, it is important that the method is able to discriminate between ions (and their ligand complexes) and particles. The techniques with this ability are synchrotron-based techniques (XANES) [37], hyperspectral imaging, SP-ICP-MS and AF4-ICP-MS [35] and SIMS-based techniques [40]. Among them, SP-ICP-MS has proven to be a good choice due to its high throughput and the possibility to discriminate between ions and particles (Table 1A).

### 2.2. Throughput

Knowing the throughput of methods is important to assess the feasibility of planned work. We evaluated the throughput of techniques according to (i) the time input for sample preparation, (ii) thevtime input for data acquisition and processing, as well as (iii) the method accessibility. The time demand for sample preparation was evaluated based on the number and duration of required steps, such as drying, freezing, chemical fixation, acid digestion and processing (slicing, homogenizing, contrasting, and staining). Methods with the highest time input needed for sample preparation are TEM-EDX, FIB-SEM-EDX and spectroscopy-based techniques (LA-ICP-MS, XANES, XAS, XRF, SIMS and PIXE) (Table 1B). Methods differ significantly in time input needed for data acquisition and processing also. The most time consuming data acquisition and processing are required for XANES, XAS, XRF, and SIMS-based methods as well as LA-ICP-MS and DFOMS-based imaging. The least accessible methods are those dependent on large synchrotron facilities because they usually require pre-application for beam line, with no guarantee of selection, plus expert data processing and interpretation. After estimating the time input and accessibility of methods we estimated the final throughput as *high*, *medium* and *low*. We estimate that AAS, ICP-MS, SP-ICP-MS and AF4-ICP-MS have the highest throughput (and accessibility). Medium throughput methods are those enabling tissue/cellular microscopy and spectroscopy, while the lowest throughput is offered by synchrotron-based techniques (Table 1B).

## 3. Interpretation of Biological Fate Data for Ag NPs

We designed an interpretation scheme for biological fate data of metal-based NPs with dissolution potential, illustrated using the literature data for Ag NPs (Figure 1). The aim of the scheme is to show links among the various research questions, available methods and the appropriate data interpretation, i.e., what can be concluded on the basis of the so-acquired data. The available Ag biological fate data for body burden are discussed separately from that on body tissue distribution/cellular internalization, as the methods for the former are primarily those categorized as quantification methods in Table 1A, while those for tissue distribution/internalization are primarily those identified as visualization methods in Table 1A.

### 3.1. Body Burden

The most commonly applied techniques to study Ag NP body burden are AAS, ICP-MS, and radioactive labelling (Table S1, Figure 2). These techniques do not provide data on metal speciation; therefore the obtained results do not confirm the presence of exclusively Ag NPs in the body. Measured values represent total Ag, including Ag NPs, their dissolved Ag^+^, Ag NPs-Ag^+^ complexes and/or Ag^+^-organic and inorganic ligand complexes associated with, or internalized by the organism [6]. Recently, promising techniques for quantifying the body burden of NPs separately from ions are SP-ICP-MS and AF4-ICP-MS [41]. The latter two are efficient methods for discrimination and quantification of Ag NPs and other Ag species, but both have serious limitations regarding size detection limits, with the lower limit being estimated as ~20 nm for Ag NPs [2,41]. These methods also require prior knowledge of the specific metal being assessed for, and the approaches required to digest and clean-up the samples may lead to misidentification of specific elements (our personal experience). One aspect to be considered in terms of Ag NP body burden is the contribution of adsorbed material in the digestive system and particularly adsorption to the body surface, which are not formally internalized but are detected as part of the total body burden. It remains a point of discussion as to whether this constitutes the bioaccumulated fraction of NPs.

### 3.2. Body Distribution

A number of different techniques have previously been applied to study the cellular internalization and body distribution of Ag NPs (Table S2, Figure 2). The majority of published data do not actually confirm the occurrence of cellular entrance and body distribution of Ag NPs, despite claims to this effect. Most of the frequently applied techniques do not discriminate between ions and particles, and thus most literature reports only confirm the presence of Ag, but not its uptake form. In addition, the formation of secondary Ag NPs from internalized Ag^+^ may lead to false positive results on cellular internalization of Ag NPs. In this case the size, shape, composition and density of re-precipitated particles compared to the engineered ones differ and should be compared by TEM and X-ray diffraction analyses. Secondary formation of Ag particles in vivo could be confirmed by exposure of organisms to ionic Ag only.

On the other hand, synchrotron-based techniques (XANES), hyperspectral imaging, SP- and AF4-ICP-MS and TOF-SIMS enable the differentiation between ions and particles and are therefore appropriate to study NP distribution. Using these approaches, Ag NP distribution inside organisms was reported in some organisms [15,42,43,44]. Endocytosis is the most frequent explanation for cellular entry of solid particles and has previously been reported. For example, Ag NPs were found in food vacuoles in the protozoan *Tetrahymena thermophila* [17], in alveolar macrophages [45], and in the vacuoles of the freshwater mixotrophic unicellular alga *Ochromonas danica* [46]. In the polychaete *Nereis diversicolor*, Ag NPs were found in the endocytic pits and endosomes of gut epithelia [47]. Ag NPs could enter the cell also when the cell membrane is damaged [15,32,43,48]. For example, Ag NP entry into fish gill [49] and plant [15] cells have been explained as transport via damaged cell membrane. Many studies report Ag NPs in different body regions without being specific about whether Ag NPs entered cells or not. For example, NPs could enter the organism through openings to the external environment, such as vulva, stomata, and chorionic pores. For example, Ag NPs were found inside zebrafish *D. rerio* embryo via transport through the chorionic channels, which was confirmed by real-time in vivo imaging using scanning near-field optical microscopy [44]. In *C. elegans* Ag NPs were found predominantly around the uterine tissue which implies the entrance of NPs through an external opening, i.e., the vulva [21,22]. In plants, Ag NPs were found to follow cuticular and stomatal pathways inside leaves [15]. Geisler-Lee et al. [14] found Ag NPs apoplastically transported through the cell wall and Ag NPs aggregated on the external part of plasmodesmata. They suggest that Ag NPs were most likely trapped by the special cell wall architecture and were not convinced that Ag NPs could enter the cell cytoplasm through the cellular membrane. Recently, the uptake of polystyrene NPs was reported for *Daphnia magna* embryos, because the brood chamber is open and in direct contact with the ambient water [50].

Taken together, these considerations imply that further studies on NPs body distribution and cellular internalization should consider two important aspects in terms of interpretation of the obtained data. Firstly, there are serious limitations in terms of techniques utilized to confirm internalization of intact NPs and to discriminate between ions and particles, as well as to discriminate between the uptake of intact NPs from the external environment and the secondary precipitation of particles from internalized ions. The latter could be effectively resolved by employing the corresponding metal salt control. Even more important is the fact that physiological interpretation of observed NP internalization is rarely provided which may result in an inaccurate understanding of the obtained data. For example, endocytosis, in particular phagocytosis, is a common physiological mechanism used by many protists or cells of multicellular organisms to acquire nutrients or to serve as an important defense mechanisms against infection by microorganisms (e.g., bacteria) and the process of removing cellular debris (e.g., dead tissue cells and old proteins etc.). The presence of NPs in certain types of cells (for example macrophages, haemocytes) therefore does not necessarily point to potential hazard for the organism, but may suggest the organism is capable of processing the NPs for removal or degradation. Also, there is no convincing evidence that the internalized NPs, especially those with high dissolution potential, bioaccumulate inside the body of organisms after passing through the epithelial barriers. Analogously, the fact that NPs were found inside the organism after passage through an opening to the external environment (e.g., vulva, stomata or chorionic pore as evidenced before) does not necessarily mean that they have passed the epithelial barriers and accumulated in the body.

## 4. Final Conclusions and Outlook

Our review shows that most of the evidence gathered to date for Ag NP body burden actually assesses total Ag, including Ag^+^, Ag NPs and Ag NPs-Ag^+^ complexes rather than confirming uptake of intact Ag NPs by organisms commonly used in environmental studies. Although not included in our analysis due to the sheer volume of publications, it is reasonable to assume that the same conclusion would be drawn from data gathered on other test systems including bacteria, yeast, protozoa and mammalian cell lines, and for other soluble or partially soluble NPs. On the other hand, many reports of internalized Ag NPs that were supported by appropriate methodology were lacking a comprehensive explanation of the possible uptake/translocation route(s). Two important aspects to be included in the interpretation of biological fate of Ag and other metal and metal oxide NP data are: limitations of the techniques utilized, the physiological background or mechanism of the observed NPs internalization, and the discussion of uptake should be grounded in consideration of the potential implications for bioaccumulation and/or hazard.

This review categorizes methods for studying the biological fate of NPs, according to their performance in terms of answering questions regarding body burden and body distribution as well as throughput and accessibility of the analytical methodology. Although we focused our review on Ag NPs, being among the most studied NPs, our conclusions regarding appropriate method selection according to the scientific aim in question can be readily applied to other metal and metal oxide NPs. There is currently no single technique that would discriminate between ions and NPs (including discriminating between the engineered variants and secondary particles precipitated in situ by organisms), and at the same time enable localization and quantification of NPs up to cellular level. We suggest that for each NP investigated, the selection of technique should be based on the characteristics of the NPs (e.g., their size, as this is a limiting factor for several methods), be driven by the aim of the study and further users of data/results (modeling, generation of a database, scientific knowledge on organism or cell biology and physiology), and include complementary methods capable of quantification and visualization of localization as well as appropriate control experiments to rule out confounding factors. For example, in the case of bioaccumulation, food chain transfer and modeling studies, high throughput analyses are of vital importance. Data should be robust enough to be further used by experts with non-biological backgrounds. For precise tissue and cell localization, high resolution imaging and discrimination among ions and particles (engineered versus in situ precipitated) are of prime importance. These data have to be interpreted by experts with knowledge on the specific organism’s anatomy, physiology and particular feeding strategy as well as the ecology of the investigated species.

At the present state of knowledge it appears that the biological fate of metal and metal oxide NPs could partly be the same as the fate of other metal species (i.e., ionic fraction) and partly that of microorganisms, cell debris, and other foreign bodies entering the organism (i.e., phagocytosis and destruction), although the relative proportions will depend on the specifics of the NP preparation and its ratio of ions to NPs under the specific environmental conditions. However, there are also particle-specific characteristics of NPs which need to be taken into consideration when their biological fate is studied. Among them are the adsorption to organism body surfaces in particular, and the transformation of NPs within the organism body following uptake. In the future, some new methodological approaches might complete our understanding of NP biological fates. For example, analysis of gene expression may indicate processes in the cell facilitating metal ion precipitation or synthesis of in situ particles. With subsequent gene knock-out of the identified processes, it might be possible to better determine the origin of intracellular NPs.

## Figures and Tables

**Table 1:** The description of techniques used for in vivo investigations of NPs’ biological fate. We present the basic characteristics of the techniques (Table 1A) in terms of their quantification and visualization properties (+ means the method can do this, - means the method does not provide this information). The throughput of methods is evaluated based on the time input for sample preparation, time input for data acquisition and processing and the accessibility of the methods (Table 1B).

**(A) Characteristics of Techniques**	**AAS, ICP-MS (OES)**	**SP-ICP-MS, AF4-ICP-MS**	Tracing Labelled NPs	**LA-ICP-MS**	**XANES**, XAS	XRF	SIMS	**PIXE**	**FIB-SEM-EDX**	**TEM-EDX**	**Hyperspectral Imaging**	DFOMS
Quantification in decomposed tissue	+	+	+	-	-	+	+		-	-	-	-
Power to quantify Ag NPs in the sample	-	+	-	-	+	-	-	-	-	-	-	-
Power to discriminate between Ag ions and NPs	-	+	-	-	+	-	+	-	-	-	+	-
Visualization on tissue slices	-	-	+	+	+	+	+	+	+	+	+	+
Visualization of Ag species at the tissue level	-	+	+	+	+	+	+	+	+	+	+	+
Visualization of Ag species at the subcellular level	-	-	+	-	+	+	+	+	+	+	-	-
**(B) Throughput**	**AAS, ICP-MS (OES)**	**SP-ICP-MS, AF4-ICP-MS**	Tracing Labelled NPs	**LA-ICP-MS**	**XANES**, XAS	XRF	SIMS	**PIXE**	**FIB-SEM-EDX**	**TEM-EDX**	**Hyperspectral Imaging**	DFOMS
Time input for sample preparation *	1	1	2	2	2	2	2	2	2	3	0	0
Time input for data acquisition; processing ^†^	0	0	0	2	3	3	3	1	1	1	2	2
Difficulty to access ^‡^	0	0	1	1	1	1	1	1	1	0	1	1
Final score ^$^	1	1	3	5	6	6	6	4	4	4	3	3
**Throughput ^&^**	***high***	***high***	***medium***	***low***	***low***	***low***	***low***	***medium***	***medium***	***medium***	***medium***	***medium***

* **Time input for sample preparation** is categorized into low (grade 0), medium (grade 1), high (grade 2) and very high (grade 3) based on the number and difficulty of sample preparation steps, including: fixation, sectioning, polishing and/or coating, and acid digestion of biological sample. ^†^ **Time input for data acquisition and/or processing**: estimated as low (grade 0), medium (grade 1), high (grade 2) and very high (grade 3) based on the time needed to obtain the signal and time to process the signal. ^‡^ **Difficulty to access**: infrastructure dependent equipment was estimated as easy (grade 0) or difficult (grade 1). ^$^ **Final score** is the sum of scores given to the first three categories. ^&^ **Throughput** was estimated based on the final score. Grades 1–2 are considered *High*, grades 3–4 *Medium* and 5–6 *Low* throughput.

## Supplementary Materials

The following are available online at http://www.mdpi.com/1996-1944/11/6/957/s1, Table S1: Existing data on the Ag NPs body burden grouped according to the techniques used. Organisms are sorted according to taxonomic position; Table S2: Review of studies on the body distribution and cell/tissue internalization of Ag NPs in organisms frequently used in environmental studies.

## Abbreviations

AAS	atomic absorption spectroscopy
AF4-ICP-MS	asymmetric flow field-flow fractionation inductively coupled plasma mass spectrometry
DFOM	dark field optical microscopy
DFOMS	dark field optical microscopy and spectroscopy
EDX	energy dispersive X-ray
FIB-SEM-EDX	focused ion beam scanning microscopy coupled with energy-dispersive X-ray spectroscopy
ICP-MS	inductively coupled plasma mass spectrometry
ICP-OES	inductively coupled plasma optical emission spectroscopy
LA-ICP-MS	laser ablation inductively coupled ion mass spectrometry
PIXE	proton induced X-ray emission
SEM	scanning electron microscopy
SIMS	secondary ion mass spectrometry
ToF-SIMS	time of flight-secondary ion mass spectrometry
SP-ICP-MS	single particle inductively coupled plasma mass spectrometry
STEM	scanning transmission electron microscopy
TEM	transmission electron microscopy
TEM-EDX	transmission electron microscopy coupled with energy-dispersive X-ray spectroscopy
XANES	X-ray absorption near edge structure spectroscopy
XAS	synchrotron X-ray absorption spectroscopy
XRF	X-ray fluorescence
