# Supplementary material for: The Biological Fate of Silver Nanoparticles from a Methodological Perspective
Source: Materials (Basel). 2018 Jun 5;11(6):957. doi: 10.3390/ma11060957 (PMC6025118; doi:10.3390/ma11060957)
Supplement: Supplementary file 1 [file materials-11-00957-s001.pdf]

Supplementary Information

The Biological Fate of Silver Nanoparticles from a Methodological Perspective

Damjana Drobne [1], Sara Novak [1], Iva Talaber [1,*], Iseult Lynch [2] and Anita Jemec Kokalj [1]

[1] Department of Biology, Biotechnical Faculty, University of Ljubljana, Ljubljana 1000, Slovenia; damjana.drobne@bf.uni-lj.si (D.D.); sara.novak@bf.uni-lj.si (S.V.); anita.jemec@bf.uni-lj.si (A.J.K.)
[2] School of Geography, Earth and Environmental Sciences, University of Birmingham, Birmingham B15 2TT, UK; I.Lynch@bham.ac.uk
[*] Correspondence: iva.talaber@bf.uni-lj.si; Tel.: +386-1423-3378

Received: 26 April 2018; Accepted: 31 May 2018; Published: date

Abbreviations:

AAS:	atomic absorption spectroscopy
AF4-ICP-MS:	asymmetric flow field-flow fractionation inductively coupled plasma mass spectrometry;
CARS:	coherent anti-Stokes Raman spectroscopy;
DFOM:	dark field optical microscopy;
DFOMS:	dark field optical microscopy and spectroscopy;
EDX:	energy dispersive X-ray;
EXAFS:	X-ray absorption fine structure spectroscopy;
FAAS:	flame atomic absorption spectroscopy;
FIB-SEM-EDX:	focused ion beam scanning microscopy coupled with energy-dispersive X-ray spectroscopy;
FIB-SIMS:	focused ion beam secondary ion mass spectrometry;
GF-AAS:	graphite furnace atomic absorption spectroscopy;
HAADF-STEM:	high angle annular dark field scanning transmission electron microscopy;
HEDFM:	hyperspectral-enhanced dark field microscopy;
ICP-MS:	inductively coupled plasma mass spectrometry;
ICP-OES:	inductively coupled plasma optical emission spectroscopy;
ICP-OES:	inductively coupled plasma optical emission spectroscopy;
LA-ICP-MS:	laser ablation inductively coupled ion mass spectrometry;
MSIS:	multispectral imaging system;
Nano-SIMS:	nanoscale secondary ion mass spectrometry;
PIXE:	proton induced X-ray emission;
μ-PIXE:	micro-proton induced X-ray emission;
SAED:	selected area electron diffraction;
SEM:	scanning electron microscopy;
SIMS:	secondary ion mass spectrometry;
SP-ICP-MS:	single particle inductively coupled plasma mass spectrometry;
STEM:	scanning transmission electron microscopy;

TEM:	transmission electron microscopy;
TEM-EDX:	transmission electron microscopy coupled with energy-dispersive X-ray spectroscopy;
ToF-SIMS:	time of flight-secondary ion mass spectrometry;
XANES:	X-ray absorption near edge structure spectroscopy;
XAS:	synchrotron X-ray absorption spectroscopy;
μ-XAS:	micro synchrotron X-ray absorption spectroscopy;
XRF:	X-ray fluorescence;
μ-XRF:	micro X-ray fluorescence;

Chapter 1

We present a detailed review of studies on the biological fate of Ag NPs in most representative organisms used in environmental studies. Table S1 presents existing data on the Ag NPs body burden and Table S2 on the body distribution and cell/tissue internalization of Ag NPs in organisms.

Table S1. Existing data on the Ag NPs body burden grouped according to the techniques used. Organisms are sorted according to taxonomic position.

**Atomic Absorption and Inductively Coupled Plasma Spectrometry**		
**Test organism**	**Reported body region for Ag NPs burden**	**Reference**
Water flea *Daphnia magna*	Total body	[1–5]
Brine shrimp *Artemia sp.,*	Total body	[6]
Marine barnacle larvae *Balanus amphitrite*	Total body	[7]
Freshwater snail *Lymnaea stagnalis*	Total body	[8,9]
Freshwater snail *Potamopyrgus antipodarum*	Total body, soft tissue, shell	[10]
Freshwater mussel *Dreissena polymorpha*	Soft tissue and different subcellular fractions; metal-rich granules (MRG), cellular debris, organelles	[11]
Estuarine snail *Peringia ulvae*	Total body	[9]
Marine snail *Littorina littorea*	Gill, kidney, stomach and visceral mass	[12]
Marine snail Crepidula onyx	Total body	[7]
Marine mussel *Mytilus galloprovincialis*	Gills, digestive glands	[13,14]
Marine mussel *Macoma balthica*	Soft tissue and shell of clams	[15]
Marine mussel *Scrobicularia plana*	Total body	[16]
Terrestrial snail *Achatina fulica*	Total body	[17]
Marine worm *Platynereis dumerilii*	Total body	[18]
Marine worm *Nereis diversicolor*	Total body	[19]

Marine worm *Hediste diversicolor*	Total body	[16]
Freshwater worm *Capitella capitata*	Total body, soft tissue, shell	[10]
Freshwater worm *Hydroides elegans*	Total body	[7]
Earthworm *Enchytraeus crypticus*	Total body	[20]
Earthworm *Eisenia fetida*	Total body	[21–24]
Earthworm *Lumbriculus variegatus*	Total body	[5]
Earthworm *Lumbricus rubellus*	Total body	[25,26]
Nematode *Caenorhabditis elegans*	Total body burden	[27]
Springtail *Folsomia candida*	Total body	[28]
Springtail *Lobella sokamensis*	Total body	[23]
Caterpillar *Achaea Janata* L.<br>Armyworm *Spodoptera litura*	Total body	[29]
Midge *Chironomus sp.*	Total body	[30]
Common carp *Cyprinus carpio*	Liver, intestine and gallbladder	[31,32]
Rainbow trout *Oncorhynchus mykiss*	Liver, kidney, gills and muscle	[33,34]
Atlantic salmon *Salmo salar*	Gills	[35]
Japanese medaka *Oryzias latipes* embryos	Total embryo	[36]
Zebrafish *Danio rerio* embryos	Total body	[6,37,38]

Zebrafish *Danio rerio* larvae	Total larvae	[39]
Zebrafish *Danio rerio* adults	Gills and eviscerated carcass	[40]
Mosquitofish *Gambusia holbrooki*	Total body	[30]
Trout *Oncorhynchus mykiss* gill cell line	Cell	[41]
Unicellular alga *Chlamydomonas reinhardtii*	Total and intracellular silver content	[42]
Unicellular alga *Euglena gracilis*	Cell	[41]
Duckweeed *Spirodela polyrhiza*	Plant tissue (not further specified)	[43,44]
Rice *Oryza sativa*	Plant tissue (not further specified)	[45]
Wheat *Triticum aestivum L.*	Roots and shoots	[46–48]
Alfalfa *Medicago sativa*	Total body	[49]
Soybean *Glycine max*	Roots and shoots<br>cell walls, organelles, and vacuoles	[47]
Oilseed crop *Crambe abyssinica*	Root tissue	[50]
Ryegrass *Lolium multiflorum*	Root tissue	[51]
Cress *Arabidopsis thaliana*	Total body, roots and shoots	[52–54]
Poplar *Populus deltoides × nigra*	Total body, roots and shoots	[53]
Tomato *Lycopersicon esculentum* seedlings	Total body	[55]
Mesocosm including different plants ant and animals (*Juncus effuses, Carex*	Total body	[30]

lurida, Panicum virgatum, and *Lobelia cardinalis*		
**Single Particle Inductively Coupled Plasma Spectrometry**		
Earthworm *Lumbriculus variegatus*	Total body (Homogenate)	[56]
Soybean *Glycine max*<br>Rice *Oryza sativa L.*	Leaves	[57]
Cress *Arabidopsis thaliana*	Root and shoot tissue<br>(middle lamella and cell walls in root tissue<br>and leaves)	[58]
**Radioactive Labeling**		
Water flea *Daphnia magna*	Gut and in the whole body	[59,60]
Harlequin fly *Chironomus* sp. larvae	Whole body	[61]
Iceland scallop *Chlamys islandica*	Digestive system, gills, mantle, kidney and muscle	[62]
Earthworms *Eisenia fetida*	Total body	[63]
Medaka fish *Oryzias latipes*	Liver, gills, kidney, heart, brain, spleen, gall bladder and intestine	[64]
Zebrafish *Danio rerio*	Whole body	[61]

Table S2. Review of studies on the body distribution and cell/tissue internalization of Ag NPs in organisms frequently used in environmental studies. Studies are listed according to the methods of detection.

Test Organism	Description of Ag NPs	Method of Detection	Results	Explanation of NPs Internalization‖	Reference
Waterflea *Ceriodaphnia cornuta*	Synthesized, Green synthesis	LM	Ag NPs accumulate in the gastrointestinal tract.	Ingestion.	[65]
Fish *Barbonymus gonionotus*	Ag NP powder , Dongyang (HK) International Group Limited, 84.60±14.38 nm†	LM	Ag NPs accumulate in tissues and internal organs.	No explanation provided.	[66]
Protozoan *Tetrahymena thermophila*	Ag NPs , Sigma-Aldrich (Buchs, Switzerland), 02 mg/mL suspensions in aqueous buffer containing sodium citrate as a stabilizer 20 nm*, 30 nm¶	HEDFM	During the 2 h of exposure, protozoan food vacuoles became filled with aggregates of Ag NPs Internalization of NPs was confirmed by observing different focal planes.	Phagocytosis.	[67]
Nematode *Caenorhabditis elegans*	Synthesized, citrate-coated, 25 nm‡ (EPA moderately hard reconstituted water)	HEDFM	Ag NPs remained predominantly in the gut; the authors imply that they may also be detected in other tissues – region not specifically described.	No direct documentation of internalization.	[68]
Fish larvae *Oryzias latipes*	NanoAmor, all spherical, a.) PVP coated, 10 nm*, 50.72 nm‡,# ; b.) PVP coated, 50 nm*, 133.15 nm‡,#; c.) Citrate-coated, 7 nm*, 13.84 nm‡,#; d.) Gum arabic coated, 6 nm*, 96.21 nm‡,#	HEDFM	Random distribution throughout the body. All Ag NPs were found in gill, gut lumina, mid-brain and liver parenchyma. No Ag NPs were found in epidermis, spine, skeletal muscle, kidney or gonad.	Possible entry due to local tissue necrosis or via endocytosis.	[69]
Protozoan *Tetrahymena thermophila*	Ag NPs , Sigma-Aldrich (Buchs, Switzerland), sodium citrate as a	HEDFM	Ag NPs accumulated in food vacuoles.	Phagocytosis is suggested as an uptake mechanism.	[67]

	stabilizer; 20 nm*, 30 nm¶				
Earthworm *Lumbriculus variegatus*	Luna PVP Ag NPs 24 ± 5 nm†; nanoComposix 30 ± 3 nm†; and 68 ± 5 nm†; NanoDyanmics 46 ± 15 nm† and 67 ± 22 nm†	HEDFM	Ag NPs detected only in the intestinal tract, but no NPs translocation confirmed.	NPs are not translocated through the intestinal barrier.	[56]
Wheat *Triticum aestivum*	Attostat, 10 nm*, 7.4 nm and 60.8 nm‡ (dH_2O), -37 mV¶	TEM	Electron-dense material was observed in shoots, but Ag was not confirmed. Ag NPs aggregates were also found when exposed to Ag salt.	Internalization due to local necrosis of tissue§.	[48]
Lepidopterans *Achaea janata L, Spodoptera litura*	Sigma Aldrich, PVP coated, < 100 nm*, 219.5 nm‡ (dH_2O), 22.3 ± 5.78 mV¶	TEM	Electron-dense material in larval gut cells. Damage of the tissue evidenced. Ag was not confirmed.	Internalization due to local necrosis of tissue§.	[29]
Unicellular freshwater alga *Chlorella vulgaris*	Ag NPs, nanoBEE consortium (University of Birmingham) PVP, PEG and citrate coated, 10 nm*	TEM	Ag NPs were localised in starch granules within the chloroplast.	Authors suggest passive passage through cell wall, which may be enhanced when cells are exposed to a high concentration of Ag NPs.	[2]
Cress *Arabidopsis thaliana*	Sigma–Aldrich (USA) 10 nm*	TEM	Ag NPs accumulate predominantly at the middle lamella and cell walls in root tissue and some Ag NPs can be translocated toward the leaves.	Authors suggest the intercellular passage of NPs through the cell wall.	[58]
Isopod *Porcellio scaber*	Sigma Aldrich, spherical, non-coated, <100 nm*, 30-200 nm† (dH_2O),	TEM EDX	No Ag NPs were found in the digestive gland S-cells.	Confirmed internalization of Ag ions only.	[70]
Fish *Oncorhynchus mykiss*	Nanostructured and amorphous materials, spherical, 49 ±18.5 nm†, 589 ± 101 nm‡, -12.52 ± 2.7 mV¶	TEM EDX	Aggregates associated with pavement cells at the gill surface. Ag was not detected by EDX.	No internalization.	[33]
Marine worm *Nereis diversicolor*	Citrate-capped, 30 ± 5 nm† (artificial estuarine water), -	TEM EDX	Aggregates in the gut lumen, close to the microvilli, and in the extracellular matrix	Internalization due to endocytosis.	[71]

	15 ± 2 mV¶		(endocytic pits and endosomes).		
Terrestrial plants *Phaseolus radiates*, *Sorghum bicolor*	ABC Nanotech, spherical, 5-25 nm†	TEM EDX	Ag NPs aggregates found in root tissue.	Internalization due to local necrosis of tissue§.	[72]
Marine mussel *Mytilus galloprovincialis*	European Joint Research Centre (Ispra, Italy), Maltose–stabilised Ag NPs 20, 40, 100 nm*	TEM EDX	Ag NPs of the same size as the source were not identified within cells.	No evidence of Ag NPs internalization. Possibility of secondary NPs formation from released Ag^+ is suggested.	[14]
Earthworm *Eisenia andrei*	NanoAmor PVP coated: 20 nm*, NanoComposix Inc., PVP coated 38.6 ± 9.8 nm†	TEM EDX	Ag NPs were found within vacuoles of the tissue.	Cross sections of body segments were analyzed. Authors suggest the Ag NPs earthworm uptake, but the mechanism is not discussed.	[22]
Alfalfa *Medicago sativa*	PVP coated Ag^0 NPs (6.3 nm†) and Ag_2S NPs (7.8 nm†) were synthesized by the authors	TEM EDX XRF	Ag NPs mainly accumulated in the (columella) border cells and elongation zone of roots. Ag_2S NPs remained largely adhered to the root exterior.	Direct uptake of small or partially dissolved NPs into the root apoplast is suggested. Dissolved Ag^+ uptake and secondary formation of Ag NPs is evidenced.	[49]
Cress *Arabidopsis thaliana*	Ted Pella, 20 nm*, 40 nm and 80 nm†, ~1 μm‡ (Hoagland media after 7 h)	STEM EDX	Ag NPs in border cells, root cap, columella and columella initials. Aapoplastic transport of Ag NPs in cell wall and at plasmodesmata. Ag NPs were also detected with Ag salt.	Ag NPs trapped by cell wall architecture. Internalization due to local necrosis of tissue§. Ag NPs secondary formation.	[54]
Unicellular freshwater alga *Raphidocelis subcapitata*	nanoComposix, 10 and 60 nm*, polyethylenimine and tannic acid coating	DFOM SEM Nano-SIMS	The majority of the Ag NPs or their dissolution products were localized around the algal cell walls. Some Ag NPs entered and deposited inside the cells beyond the periplasmic space.	No explanation provided.	[73]

Lettuce *Lactuca sativa*	PPG, uncoated, round-shaped, 38.6 nm†, 47.9± 29.2 nm (test media) ‡, -29.5 mV¶	SEM<br>EDX<br>μ-XRF<br>XANES<br>ToF-SIMS	Ag NPs found in the cuticle, between the guard cells, the sub-stomatal chamber, the main vein and the cell wall thickening. Ag NPs aggregates were also found with Ag salt exposure.	Cuticular and stomatal pathways of penetration. Local tissue necrosis evidenced. Ag NPs secondary formation.	[74]
Fish embryo *Fundulus heteroclitus*	Citrate-coated, pseudo-spherical, 3 nm†; -30 mV¶ at pH 7.5, 0.3-20 nm‡ (10% ASW$)	μ-XRF<br>XANES	Ag NPs aggregates at the surface and inside of the embryo. Intake depends on the salinity.	Penetration through chorionic channels.	[75]
Nematode *Caenorhabditis elegans,*	PVP coated: 58.3 ± 12.9 nm†, -6.1 mV¶;Sulfidated (S-AgNPs): 64.5 ± 19.4 nm†, -28.1 mV¶	μ-XRF	Similar Ag spatial distributions found in case of PVP-Ag NPs and Ag-salt, but not in case of S-Ag NPs (Ag^+ was complexed by S). No speciation data.	No clear evidence of internalization.	[76]
Polychaete *Nereis virens*	Nanocomposix Inc., citrate coated: 29.0 ± 3.1 nm†,-41.8 mV¶, PVP coated: 28.7 ± 2.1 nm†, -26,7 mV¶	XAS<br>μ-XRF	Ag NPs detected inside the organism. Location not described. The distribution of Ag metal, AgCl, and Ag_2S species was coating dependent.	No clear evidence of cellular internalization.	[77]
Duckweed *Landoltia punctata*	PVP coated Ag^0 NPs (6.3 nm†) and Ag_2S NPs (7.8 nm†) were synthesized by the authors	μ-XRF<br>EXAFS	Nanoparticulate phases of metals readily attach to the plants. Adhered particles are taken up to some degree into the plant root vasculature, either after being solubilized or as particles.	Authors also suggest ionic uptake into the active region of the root tip and secondary formation of NPs.	[78]
Wheat *Triticum aestivum L.*	NanoAmor, PVP coated 52 ± 1 nm (determined by XRD)	μ-XRF<br>μ-XANES	Ag NPs mostly adhered to the epidermis of roots and accumulated preferentially in discontinuities between root epidermal cells. No Ag^0 was found inside roots.	Transfer of NPs due to local damage of roots. Root hairs also considered as potential points of entry of NPs, due to thin cell walls and role in nutrient acquisition. Symplastic and apoplastic transfer of Ag^+ is possible. Secondary Ag NPs were also identified.	[46]
Unicellular algae	AMEPOX; 3– 8 nm*, alkane	CARS	Large aggregates of NPs detected external	No evidence of NPs	[4]

Raphidocelis subcapitata	coating		to the algae cells.	internalization.	
Earthworm *Lumbricus rubellus*	NanoTrade Ltd 50 nm*	XANES	Ag NPs confirmed in the chloragogenous tissue, within the typhlosole, around the basal intestinal surface, nephridial tubules and near the base of setae.	Internalization to local hotspot, potentially as intact NPs, via endocytosis pathways in intestinal epithelia.	[26]
Zebrafish *Danio reiro* embryos	Ag NPs (41.6 ± 9.1 nm†) were synthesized by the authors	DFOMS	Diffusion and transport of single Ag NPs in embryos.	Penetration through chorionic pores.	[72]
Nematode *Caenorhabditis elegans*	Sigma Aldrich, <100 nm*, 20 nm†, 14-20 nm‡ (growth medium)	DFOMS	Electron-dense material found around uterine area, but Ag was not confirmed.	Penetration through vulva to reach uterine area.	[79]
Zebrafish *Danio reiro*	PlasmaChem GmbH (Berlin, Germany) 20 nm* hydrodynamic diameters > 100 nm	SIMS	Ag hot spots were found mainly around liver blood vessels and in the interstitial tissue between the intestine and the liver. Authors do not explicitly say that Ag NPs were detected, but refer to »Ag hot spots«.	Authors suggest that Ag NPs cross the skin of zebrafish larvae and accumulate in blood vessels. However, no discussion regarding the passage of dissolved Ag^+ is given.	[80]
Zebrafish *Danio reiro*	PVP and sodium citrate stabilized Ag NPs, hydrodynamic diameter 117 ± 24 nm	LA-ICP-MS	Ag NPs detected in the chorion and the perivitelline space, whereas only minor amounts reach the embryo.	Not discussed.	[81]
Zebrafish *Danio rerio* embryos	Synthesized, citrate coated, spherical, 41.6 ± 9.1 nm*, 45.5 ± 9.8 nm‡,~	DFOMS MSIS	Ag NPs found in the inner mass of embryos.	Penetration through chorionic channels.	[82]
Unicellular alga *Chlamydomonas reinhardtii*	Synthesized, 11.7 ± 1.9 nm†	Nano-SIMS HAADF-STEM XAS EDX	The observations with all listed methods collectively confirmed the internalization of Ag NPs into the periplasmic space of *C. reinhardtii.*	No explanation is provided for the internalization of Ag NPs into the periplasmic space. Possibility of secondary NPs formation	[83]

	SAED	from released Ag^{+} is suggested.

[*]Data by supplier, [†]TEM/SEM, [‡]Hydrodynamic diameter (DLS), [¶]Zeta potential, [§] according to Larue et al., 2014, [‖]this is our explanation of obtained results, [#]fish test medium after 1 h, [$]ASW: Artificial Sea Water, [~]zebrafish egg water.

Chapter 2: Supplementary Description of Methods For NPs Body Burden, NPs Body Distribution and Cellular Internalisation

Studying interactions between nanomaterials and organisms is a multi-disciplinary approach where experts in biology, physiology and nanomaterials should be complemented with understanding of the performance of instruments and data processing. There are several review papers presenting techniques appropriate for nanomaterials detection in biological samples [84–87]. However, they are mostly focused on technical characteristics of those methods, but much less on biological nature of a sample and the purpose of using these techniques. In this section we prepared a detailed review of characteristics of the methods applied to study the biological fate of NPs. First, we briefly describe the basic principle of each method. The main focus is to describe the specificity of each method to separate NPs from ions. The limitations of each method are discussed along. These data were the basis to extract the summary of methods characteristics presented in the main body of the manuscript (**Table 1, Figure 1**).

2.1. Atomic absorption spectrometry (AAS) and inductively coupled plasma spectrometry (ICP-MS)

Basic principle: The basic difference between AAS and ICP-MS is that AAS relies on the atomic absorption process while ICP-MS is an atomic/ionic emission spectroscopic technique. A combustion flame or graphite furnace is typically used for AAS while ICP uses plasma for atomic or ionic species generation. Detection limit by ICP is more than three orders of magnitude lower than by AAS [88]. Most frequently used techniques for elemental analyses of animal or plant tissue include flame atomic absorption spectroscopy (FAAS), graphite furnace atomic absorption spectroscopy (GF-AAS), inductively coupled plasma optical emission spectroscopy (ICP-OES) and inductively coupled plasma mass spectrometry (ICP-MS).

Specificity: All of the above techniques do not discriminate between ions and particulate matter as they are applied to decomposed samples. Both AAS and ICP-MS can provide data for tissue level if enough tissue is available for separate analyses of different body parts.

Limitations: Biological matrices contain abundant organic matter that must be oxidized prior to analysis to release the bound metal and reduce the physical interference from solid matter during analysis. For this purpose, acid digestion and microwave heating are usually used [89]. During this pre-analysis step, NPs are decomposed, and element of interest is released [90]. Discrimination of different chemical species is thus not possible.

2.2. Single particle inductively coupled plasma mass spectrometry (SP-ICP-MS)

Basic principle: Liquid sample is introduced into the ICP-MS instrument by using a nebulization system, which produces an aerosol of polydisperse droplets. Once the droplets are in the plasma, the solvent evaporates forming solid particles, which in turn are vaporized and their elements atomized and ionized. Ions are extracted through the interface into the mass spectrometer, where they are separated according to their mass/charge ratio and detected [58,91]. The intensity of each pulse is proportional to the mass of the element (i.e. number of atoms) in

each detected NP [91]. This method is suited for the analysis of body, organ/tissue NP burden and allows a simultaneous determination of elemental chemical composition, concentration, number concentration, size, and the number size distribution of NPs [91].

Specificity: SP-ICP-MS can differentiate the particle of interest from other incidental particles of the same size, but of different composition [92].

Limitations: SP-ICP-MS is not able to distinguish among particles, aggregates and agglomerates. It is particularly suitable for NPs consisting of one element only and sizes higher than 20 nm [92]. At the moment, the application of this method for biological samples is still under development and a number of questions need to be resolved. An important aspect is how to store the samples prior to analysis to avoid NPs transformation. It needs to be considered that approaches required to digest and clean-up the samples may lead to misidentification of specific elements and may cause transformation of NPs (our personal experience, unpublished data).

2.3. Asymmetrical flow field-flow fractionation inductively coupled plasma mass spectrometry (AF4-ICP-MS)

Basic principle: AF4-ICP-MS is a separation technique where metal species are size-separated in a thin open channel with laminar flow under the influence of a perpendicular external field (separation force). Separation system is based on asymmetrical flow field of particles [92]. The elemental analysis is done in the ICP-MS. This technique is also suitable for body, organ/tissue burden analyses and has a better resolution than the SP-ICP-MS. The enrichment of the nanoparticle fraction and simultaneous reduction of the ionic background via AF4 results in a clearly improved ICP-MS detection sensitivity, which enables a more refined identification and size characterization of the migrated ion species.

Specificity: It gives mass and element specific information and can detect smaller (2-50 nm) particles than SP-ICP-MS. As all ICP-MS, it has element specific capabilities, including mixed metals analysis [92]. Coupling of AF4 to SP-ICP-MS results in improved SP-ICP-MS sensitivity. AF4 and SP-ICP-MS are used to determine number and size of NPs in a mixture. In this combination, AF4 is essential for providing sample sub-streams that are sufficiently purified and simplified for the SP-ICP-MS analysis [93].

Limitations: There may be difficulties in sample separation due to particle aggregation within the channels and particle interactions with the membrane of the analyser.

2.4. Tracing labelled NPs

Basic principle: Nanoparticles can be labelled with radioactive isotopes, stable isotopes or florescent dyes. They are detected in tissue homogenates using spectrophotometric approach or by fluorescent or radioactive imaging [62,94]. Stable isotope labelling has been successfully used in conjunction with multiple-collector-inductively-coupled-plasma-mass-spectrometry (MC-ICP-MS) [9,95].

Specificity: Labelling of NPs with radioactive or stable isotopes is a useful tool for the highly sensitive and selective detection of NPs in the environment and organisms, thus enabling the tracing of their uptake, distribution and clearance with high sensitivity [9,95,96]. Radiolabelled Ag NPs have been prepared using different approaches, including the following: (i) *de novo* synthesis using gamma radioisotope $^{110m}AgNO_3$ as a

precursor [62,64,97], (ii) the adsorption of $^{110m}AgNO_3$ onto existing Ag NPs [59], or (iii) the neutron activation of Ag NPs [98]. Measurements can be done on tissue level and localisation can reveal the presence of Ag NPs at the cellular level (autoradiography).

Limitations: These methods can modify the NP surface chemistry and might alter its behaviour, but the general problem is that they are not able to differentiate between particulate or ionic dissolved forms of NPs after entering the tissue [12,64]. Radioactive labelling demands purpose-built laboratory and equipment. Stable isotope labelling is not beset with the problems of other methods such as fluorescent dyes or radioactive isotope labelling, where the label may be lost as a result of dissociation or radioactive decay [9].

2.5. Laser ablation inductively coupled plasma mass spectrometry (LA-ICP-MS)

Basic principle: The upper layer of a dry sample (whole organism or lyophilised slices) mounted on sample holder is sputtered by laser ablation and subsequently correlates the measured ion signals with one of the matrix elements by inductively coupled plasma mass spectrometry [99–101].

Specificity: The advantage of this method is the tissue mapping for elemental distribution, but it does not distinguish between ionic or particulate form of investigated element. The quantitative imaging by LA-ICP-MS requires the external matrix-matched calibration which is not trivial [100].

Limitations: One disadvantage of LA-ICP-MS is the occurrence of nonstoichiometric effects in the transient signals, defined as elemental fractionation. The internal standards are needed for quantitative analysis [99].

2.6. Secondary ion mass spectroscopy (SIMS)

Basic principle: Secondary ion mass spectroscopy (SIMS) is well established as a surface analytical method for advanced material research of composition of solid surfaces and thin films by sputtering the surface of the specimen with a focused primary ion beam and collecting and analysing the ejected secondary ions. In generated charged molecules or molecule fragments their mass-to-charge ratios are measured. Recent advances in the instrumentation have made these techniques even more powerful and applicable to biological research as well [102]. It utilizes a tightly focused primary ion beam to desorb chemical species from a solid matrix. A variety of different SIMS instrumentation has been used for imaging at nanoscale, among them ToF-SIMS, FIB-SIMS and Nano-SIMS [103]. In case of TOF-SIMS the detached particles are then accelerated into a flight tube and their mass is determined by measuring the exact time at which they reach the detector [104]. The main advantage of Nano-SIMS over TOF-SIMS and other generations of SIMS is the ability of Nano-SIMS to operate at nanoscale resolution, whilst maintaining both excellent signal transmission and high spatial resolution [105].

Specificity: SIMS offers information on elements present in the sample, but detailed molecular information can also be gained with high sensitivity [106]. The information on elemental composition of the sample is provided in the form of a mass spectrum and as elemental maps. An approximate range of 30-50 nm was provided for lateral resolution for ToF- and Nano-SIMS [73,103].

Limitations: The analysis of the obtained data is complex. Generally, it does not produce quantitative data. Quantification is possible with the use of standards. Optical capabilities are typically limited, which presents a difficulty for finding specific regions of interest for analysis. Charging may also be a problem in some samples

[104]. Limitations of SIMS are complex sample preparation, difficult to differentiate NP from localized ions and ultrahigh vacuum required [73,103].

2.7. Dark-field single nanoparticle optical microscopy and spectroscopy (DFOMS)

Basic principle: Dark-field light microscopy, which captures scattered light from the sample, is ideal for identifying strongly scattering objects such as Ag NPs in low scattering matrices i.e. dark background [107]. DFOMS enables imaging on tissue level on slices or even in small organisms (mm range).

Specificity: It provides *in vivo* imaging in real-time [108].

Limitations: With this method only very bright NPs can be detected - noble metals NPs, NPs with highest quantum yield (QY) of Rayleigh scattering. Very small particles cannot be detected [109]. DFOMS is known for limited spatial resolution [73].

2.8. Hyperspectral enhanced dark-field imaging (HEDFM)

Basic principle: Hyperspectral enhanced dark-field imaging is an optimized dark field microscopy. It allows tissue level or small organism (in the mm range) investigation. In case of NP imaging, an analysis is based on optical signals from resonant light scattering and a spectral signature library for NP of interest. Under enhanced dark field conditions, particles appear 150-fold brighter due to Koehler illumination and the critical illumination by a collimated light source at oblique angles [110]. The **HEDFM** spectrometer has the ability to acquire the optical spectrum for all points in a microscope image and couple it with specialized spectra [110]. In **HEDFM**, spectral information of each pixel of the image is added as a third dimension of values to the two-dimensional spatial image, generating a three-dimensional data cube, sometimes referred to as *hypercube data* or as an *image cube.* It is possible to use dark field microscopy for the detection of metallic nanoparticles, since due to their plasmonic properties NPs with dimensions larger than 50 nm scatter light strongly at a particular resonant wavelength [111].

Specificity: It is possible to distinguish between ions and NPs, because they produce different signals [69,112,113].

Limitations: Detection of very small NPs (below 50 nm) is possible, but requires increasing illumination intensity. Biological media may significantly affect the surface characteristics of NPs due to the formation of a corona, which in turn affects the signal [111]. Therefore, the effects of biological media on NPs must be well known to properly interpret the obtained spectral imaging signal.

2.9. Transmission electron microscopy coupled with energy-dispersive X-ray spectroscopy (TEM-EDX)

Basic principle: In transmission electron microscopy (TEM) a beam of electrons is transmitted through a specimen, it interacts with specimen and forms an image by emitting electrons. When a beam of electrons interacts with the sample being studied, characteristic X-rays are also emitted [48,79]. TEM can thus be used for intracellular imaging and as well as chemical analyses.

Specificity: TEM-EDX provides high lateral resolution (<10 nm) of resin embedded sections (<100 nm) [114].

Limitations: Conventional TEM, although powerful in terms of lateral resolution, is limited by low signal intensity due to thin samples (low interaction volume) [115]. Also, electron dense deposits seen in the sample may be artefacts from osmium or uranium crystals formed during sample preparation [116] or any other electron-dense cellular structure. It is therefore crucial, that EDX is applied to identify the nature of the observed spots. In the case of un-sectioned samples (for example bacteria) it is difficult to confirm that material is internalized and not adsorbed to the cell surface or any other extracellular material [117].

2.10. Focused ion beam scanning microscopy coupled with energy-dispersive X-ray spectroscopy (FIB-SEM-EDX)

Basic principle: FIB-SEM-EDX is a scanning microscope with an electron column and an ion column embedded in the same specimen chamber where both beams are aiming at the same point on the specimen surface. FIB-SEM-EDX is imaging and analytical technique which permits simultaneous sectioning, electron imaging of selected region and EDX analyses at any desired location [118,119].
Specificity: This type of microscopy allows imaging and elemental analyses of FIB exposed regions. The FIB-SEM investigation can be applied to bulk samples, prepared for conventional SEM at any chosen site or to bulk resin-embedded specimens, prepared for conventional TEM. The FIB-SEM allows the 3D imaging of NPs in a single cell, albeit at lower spatial resolution than in TEM. As a result, the absolute amount of NPs per cell could be estimated which is not possible by any other tool [120].
Limitations: FIB-SEM-EDX cannot distinguish between ions and NPs.

2.11. Particle induced X-ray emission (PIXE)

Basic principle: PIXE technique provides a multi-elemental imaging for small biological samples. A beam of protons is accelerated to an energy of a few mega-electron-volts that excites characteristic X-rays in the atoms of the specimen [121]. It allows the investigation of samples up to 50 μm thick.
Specificity: The elemental distribution and concentration at the tissue level is provided with the lateral resolution in the micron range [70]. With this method, imaging and X-rays based identification of elemental composition is possible.
Limitations: The system is not as sensitive for higher-Z elements, hence not all elements can be measured. It is also a destructive imaging technique which induces stronger beam damage to the specimen than other X-ray-induced methods described before [70,122]. Exessive damages can be avoided by using lower beam intensities.

2.12. X-ray absorption spectroscopy (XAS) and micro-X-ray fluorescence (XRF)

Basic principle: X-ray absorption micro spectroscopy (XAS) technique provides information on the chemical state of the metal-rich particles detected by **micro-X-ray Fluorescence** (XRF) mapping [123]. With both methods it is possible to generate chemical maps of an element in relation to its oxidation state and chemical bonding. X-rays are absorbed in matter and the energy of the X-rays is converted into fluorescent X-rays. The incident X-ray energy finally becomes the thermal energy of the absorber. The X-ray absorption spectra of condensed matter near the threshold energy have fine structures (**X-ray absorption near edge structure spectroscopy (**XANES)),

observable at energies less than the threshold energy. Obtained spectra show both the line shape modification and chemical shift of the absorption edge or peak [124]. With XANES, complete maps of the chemical forms can be derived, sometimes revealing details which would remain hidden using only μ-XRF and μ-XAS. Additionally, irradiation durations and the elapsed time between the start and completion of data collection for each pixel are significantly reduced [125]. Sensitivity of these methods is very high if the light source is a synchrotron [126]. Synchrotron X-rays are often preferred to desktop-generated X-rays as they offer a significantly higher resolution, a better signal-to-noise ratio, short acquisition times and quantitative reconstructions and provide phase contrast in addition to absorption contrast imaging [127].

Specificity: Besides elemental characterization, this technique enables also the analysis of the chemical (e.g. oxidation) state of the element of interest [128]. The technique provides subcellular analyses.

Limitations: Beam damage of samples can occur. Effects of radiation damage can vary from subtle changes in spectra to, in extreme cases, total sample destruction [125]. Areas within a thin specimen, which are denser or thicker, will provide a more intense signal even if the element of interest is homogeneously distributed. In the elemental map, elements can appear co-localised, when in fact could be located at different depths within the sample [126]. Another limitation is the inability to distinguish between scattering atoms with little difference in atomic number. It is difficult to use for light elements [129].

References:

1. McTeer, J.; Dean, A. P.; White, K. N.; Pittman, J. K., Bioaccumulation of silver nanoparticles into Daphnia magna from a freshwater algal diet and the impact of phosphate availability. *Nanotoxicology* **2014,** 8, (3), 305-316.
2. Kalman, J.; Paul, K. B.; Khan, F. R.; Stone, V.; Fernandes, T. F., Characterisation of bioaccumulation dynamics of three differently coated silver nanoparticles and aqueous silver in a simple freshwater food chain. *Environmental Chemistry* **2015,** 12, (6), 662-672.
3. Pakrashi, S.; Tan, C.; Wang, W. X., Bioaccumulation-based silver nanoparticle toxicity in Daphnia magna and maternal impacts. *Environmental toxicology and chemistry* **2017**.
4. Ribeiro, F.; Gallego-Urrea, J. A.; Goodhead, R. M.; Van Gestel, C. A.; Moger, J.; Soares, A. M.; Loureiro, S., Uptake and elimination kinetics of silver nanoparticles and silver nitrate by Raphidocelis subcapitata: The influence of silver behaviour in solution. *Nanotoxicology* **2015,** 9, (6), 686-695.
5. Khan, F. R.; Paul, K. B.; Dybowska, A. D.; Valsami-Jones, E.; Lead, J. R.; Stone, V.; Fernandes, T. F., Accumulation dynamics and acute toxicity of silver nanoparticles to Daphnia magna and Lumbriculus variegatus: implications for metal modeling approaches. *Environmental science & technology* **2015,** 49, (7), 4389-4397.
6. Lacave, J. M.; Fanjul, Á.; Bilbao, E.; Gutierrez, N.; Barrio, I.; Arostegui, I.; Cajaraville, M. P.; Orbea, A., Acute toxicity, bioaccumulation and effects of dietary transfer of silver from brine shrimp exposed to PVP/PEI-coated silver nanoparticles to zebrafish. *Comparative Biochemistry and Physiology Part C: Toxicology & Pharmacology* **2017,** 199, 69-80.
7. Chan, C. Y. S.; Chiu, J. M. Y., Chronic effects of coated silver nanoparticles on marine invertebrate larvae: A Proof of Concept Study. *PloS one* **2015,** 10, (7), e0132457.
8. Croteau, M.-N.; Misra, S. K.; Luoma, S. N.; Valsami-Jones, E., Silver bioaccumulation dynamics in a freshwater invertebrate after aqueous and dietary exposures to nanosized and ionic Ag. *Environmental science & technology* **2011,** 45, (15), 6600-6607.
9. Khan, F. R.; Misra, S. K.; García-Alonso, J.; Smith, B. D.; Strekopytov, S.; Rainbow, P. S.; Luoma, S. N.; Valsami-Jones, E., Bioaccumulation dynamics and modeling in an estuarine invertebrate following aqueous exposure to nanosized and dissolved silver. *Environmental science & technology* **2012,** 46, (14), 7621-7628.
10. Ramskov, T.; Forbes, V. E.; Gilliland, D.; Selck, H., Accumulation and effects of sediment-associated silver nanoparticles to sediment-dwelling invertebrates. *Aquatic Toxicology* **2015,** 166, 96-105.
11. Zimmermann, S.; Ruchter, N.; Loza, K.; Epple, M.; Sures, B., Nanoparticulate versus ionic silver: Behavior in the tank water, bioaccumulation, elimination and subcellular distribution in the freshwater mussel Dreissena polymorpha. *Environmental pollution* **2017,** 222, 251-260.
12. Li, H.; Turner, A.; Brown, M. T., Accumulation of aqueous and nanoparticulate silver by the marine gastropod Littorina littorea. *Water, Air, & Soil Pollution* **2013,** 224, (1), 1354.
13. Gomes, T.; Pereira, C. G.; Cardoso, C.; Sousa, V. S.; Teixeira, M. R.; Pinheiro, J. P.; Bebianno, M. J., Effects of silver nanoparticles exposure in the mussel Mytilus galloprovincialis. *Marine environmental research* **2014,** 101, 208-214.
14. Jimeno-Romero, A.; Bilbao, E.; Izagirre, U.; Cajaraville, M.; Marigómez, I.; Soto, M., Digestive cell lysosomes as main targets for Ag accumulation and toxicity in marine mussels, Mytilus galloprovincialis, exposed to maltose-stabilised Ag nanoparticles of different sizes. *Nanotoxicology* **2017,** 11, (2), 168-183.
15. Dai, L.; Syberg, K.; Banta, G. T.; Selck, H.; Forbes, V. E., Effects, uptake, and depuration kinetics of silver oxide and copper oxide nanoparticles in a marine deposit feeder, Macoma balthica. *ACS Sustainable Chemistry & Engineering* **2013,** 1, (7), 760-767.
16. Mouneyrac, C.; Buffet, P.-E.; Poirier, L.; Zalouk-Vergnoux, A.; Guibbolini, M.; Risso-de Faverney, C.; Gilliland, D.; Berhanu, D.; Dybowska, A.; Châtel, A., Fate and effects of metal-based nanoparticles in two marine invertebrates, the bivalve mollusc Scrobicularia plana and the

annelid polychaete Hediste diversicolor. *Environmental Science and Pollution Research* **2014,** 21, (13), 7899-7912.
17. Chen, Y.; Si, Y.; Zhou, D.; Dang, F., Differential bioaccumulation patterns of nanosized and dissolved silver in a land snail Achatina fulica. *Environmental Pollution* **2017,** 222, 50-57.
18. García-Alonso, J.; Rodriguez-Sanchez, N.; Misra, S. K.; Valsami-Jones, E.; Croteau, M.-N.; Luoma, S. N.; Rainbow, P. S., Toxicity and accumulation of silver nanoparticles during development of the marine polychaete Platynereis dumerilii. *Science of the Total Environment* **2014,** 476, 688-695.
19. Cong, Y.; Banta, G. T.; Selck, H.; Berhanu, D.; Valsami-Jones, E.; Forbes, V. E., Toxic effects and bioaccumulation of nano-, micron-and ionic-Ag in the polychaete, Nereis diversicolor. *Aquatic toxicology* **2011,** 105, (3-4), 403-411.
20. Topuz, E.; van Gestel, C. A., The effect of soil properties on the toxicity and bioaccumulation of Ag nanoparticles and Ag ions in Enchytraeus crypticus. *Ecotoxicology and environmental safety* **2017,** 144, 330-337.
21. Shoults-Wilson, W. A.; Reinsch, B. C.; Tsyusko, O. V.; Bertsch, P. M.; Lowry, G. V.; Unrine, J. M., Effect of silver nanoparticle surface coating on bioaccumulation and reproductive toxicity in earthworms (Eisenia fetida). *Nanotoxicology* **2011,** 5, (3), 432-444.
22. Velicogna, J. R.; Schwertfeger, D. M.; Jesmer, A. H.; Scroggins, R. P.; Princz, J. I., The bioaccumulation of silver in Eisenia andrei exposed to silver nanoparticles and silver nitrate in soil. *NanoImpact* **2017,** 6, 11-18.
23. Kwak, J. I.; An, Y.-J., Trophic transfer of silver nanoparticles from earthworms disrupts the locomotion of springtails (Collembola). *Journal of hazardous materials* **2016,** 315, 110-116.
24. Mukherjee, A.; Hawthorne, J.; White, J. C.; Kelsey, J. W., Nanoparticle silver coexposure reduces the accumulation of weathered persistent pesticides by earthworms. *Environmental toxicology and chemistry* **2017,** 36, (7), 1864-1871.
25. Antisari, L. V.; Carbone, S.; Gatti, A.; Ferrando, S.; Nacucchi, M.; De Pascalis, F.; Gambardella, C.; Badalucco, L.; Laudicina, V. A., Effect of cobalt and silver nanoparticles and ions on Lumbricus rubellus health and on microbial community of earthworm faeces and soil. *Applied soil ecology* **2016,** 108, 62-71.
26. Diez-Ortiz, M.; Lahive, E.; Kille, P.; Powell, K.; Morgan, A. J.; Jurkschat, K.; Van Gestel, C. A.; Mosselmans, J. F. W.; Svendsen, C.; Spurgeon, D. J., Uptake routes and toxicokinetics of silver nanoparticles and silver ions in the earthworm Lumbricus rubellus. *Environmental toxicology and chemistry* **2015,** 34, (10), 2263-2270.
27. Contreras, E. Q.; Puppala, H. L.; Escalera, G.; Zhong, W.; Colvin, V. L., Size-dependent impacts of silver nanoparticles on the lifespan, fertility, growth, and locomotion of Caenorhabditis elegans. *Environmental toxicology and chemistry* **2014,** 33, (12), 2716-2723.
28. Waalewijn-Kool, P. L.; Klein, K.; Forniés, R. M.; van Gestel, C. A., Bioaccumulation and toxicity of silver nanoparticles and silver nitrate to the soil arthropod Folsomia candida. *Ecotoxicology* **2014,** 23, (9), 1629-1637.
29. Yasur, J.; Rani, P. U., Lepidopteran insect susceptibility to silver nanoparticles and measurement of changes in their growth, development and physiology. *Chemosphere* **2015,** 124, 92-102.
30. Lowry, G. V.; Espinasse, B. P.; Badireddy, A. R.; Richardson, C. J.; Reinsch, B. C.; Bryant, L. D.; Bone, A. J.; Deonarine, A.; Chae, S.; Therezien, M., Long-term transformation and fate of manufactured Ag nanoparticles in a simulated large scale freshwater emergent wetland. *Environmental science & technology* **2012,** 46, (13), 7027-7036.
31. Gaiser, B. K.; Fernandes, T. F.; Jepson, M. A.; Lead, J. R.; Tyler, C. R.; Baalousha, M.; Biswas, A.; Britton, G. J.; Cole, P. A.; Johnston, B. D., Interspecies comparisons on the uptake and toxicity of silver and cerium dioxide nanoparticles. *Environmental toxicology and chemistry* **2012,** 31, (1), 144-154.
32. Jang, M.-H.; Kim, W.-K.; Lee, S.-K.; Henry, T. B.; Park, J.-W., Uptake, tissue distribution, and depuration of total silver in common carp (Cyprinus carpio) after aqueous exposure to silver nanoparticles. *Environmental science & technology* **2014,** 48, (19), 11568-11574.

33. Scown, T. M.; Santos, E. M.; Johnston, B. D.; Gaiser, B.; Baalousha, M.; Mitov, S.; Lead, J. R.; Stone, V.; Fernandes, T. F.; Jepson, M., Effects of aqueous exposure to silver nanoparticles of different sizes in rainbow trout. *Toxicological Sciences* **2010,** 115, (2), 521-534.
34. Joo, H. S.; Kalbassi, M. R.; Yu, I. J.; Lee, J. H.; Johari, S. A., Bioaccumulation of silver nanoparticles in rainbow trout (Oncorhynchus mykiss): influence of concentration and salinity. *Aquatic Toxicology* **2013,** 140, 398-406.
35. Farmen, E.; Mikkelsen, H.; Evensen, Ø.; Einset, J.; Heier, L.; Rosseland, B.; Salbu, B.; Tollefsen, K.; Oughton, D., Acute and sub-lethal effects in juvenile Atlantic salmon exposed to low μg/L concentrations of Ag nanoparticles. *Aquatic Toxicology* **2012,** 108, 78-84.
36. Kataoka, C.; Ariyoshi, T.; Kawaguchi, H.; Nagasaka, S.; Kashiwada, S., Salinity increases the toxicity of silver nanocolloids to Japanese medaka embryos. *Environmental Science: Nano* **2015,** 2, (1), 94-103.
37. Kim, K.-T.; Truong, L.; Wehmas, L.; Tanguay, R. L., Silver nanoparticle toxicity in the embryonic zebrafish is governed by particle dispersion and ionic environment. *Nanotechnology* **2013,** 24, (11), 115101.
38. Asharani, P.; Lianwu, Y.; Gong, Z.; Valiyaveettil, S., Comparison of the toxicity of silver, gold and platinum nanoparticles in developing zebrafish embryos. *Nanotoxicology* **2011,** 5, (1), 43-54.
39. López-Serrano, A.; Muñoz-Olivas, R.; Sanz-Landaluze, J.; Olasagasti, M.; Rainieri, S.; Cámara, C., Comparison of bioconcentration of ionic silver and silver nanoparticles in zebrafish eleutheroembryos. *Environmental pollution* **2014,** 191, 207-214.
40. Griffitt, R. J.; Lavelle, C. M.; Kane, A. S.; Denslow, N. D.; Barber, D. S., Chronic nanoparticulate silver exposure results in tissue accumulation and transcriptomic changes in zebrafish. *Aquatic toxicology* **2013,** 130, 192-200.
41. Yue, Y.; Li, X.; Sigg, L.; Suter, M. J.; Pillai, S.; Behra, R.; Schirmer, K., Interaction of silver nanoparticles with algae and fish cells: a side by side comparison. *Journal of nanobiotechnology* **2017,** 15, (1), 16.
42. Piccapietra, F.; Allué, C. G.; Sigg, L.; Behra, R., Intracellular silver accumulation in Chlamydomonas reinhardtii upon exposure to carbonate coated silver nanoparticles and silver nitrate. *Environmental science & technology* **2012,** 46, (13), 7390-7397.
43. Jiang, H. S.; Li, M.; Chang, F. Y.; Li, W.; Yin, L. Y., Physiological analysis of silver nanoparticles and AgNO3 toxicity to Spirodela polyrhiza. *Environmental Toxicology and Chemistry* **2012,** 31, (8), 1880-1886.
44. Jiang, H. S.; Yin, L. Y.; Ren, N. N.; Zhao, S. T.; Li, Z.; Zhi, Y.; Shao, H.; Li, W.; Gontero, B., Silver nanoparticles induced reactive oxygen species via photosynthetic energy transport imbalance in an aquatic plant. *Nanotoxicology* **2017,** 11, (2), 157-167.
45. Thuesombat, P.; Hannongbua, S.; Akasit, S.; Chadchawan, S., Effect of silver nanoparticles on rice (Oryza sativa L. cv. KDML 105) seed germination and seedling growth. *Ecotoxicology and environmental safety* **2014,** 104, 302-309.
46. Pradas del Real, A. E.; Vidal, V.; Carrière, M.; Castillo-Michel, H.; Levard, C.; Chaurand, P.; Sarret, G., Silver Nanoparticles and Wheat Roots: A Complex Interplay. *Environmental science & technology* **2017,** 51, (10), 5774-5782.
47. Quah, B.; Musante, C.; White, J. C.; Ma, X., Phytotoxicity, uptake, and accumulation of silver with different particle sizes and chemical forms. *Journal of Nanoparticle Research* **2015,** 17, (6), 277.
48. Dimkpa, C. O.; McLean, J. E.; Martineau, N.; Britt, D. W.; Haverkamp, R.; Anderson, A. J., Silver nanoparticles disrupt wheat (Triticum aestivum L.) growth in a sand matrix. *Environmental science & technology* **2013,** 47, (2), 1082-1090.
49. Stegemeier, J. P.; Schwab, F.; Colman, B. P.; Webb, S. M.; Newville, M.; Lanzirotti, A.; Winkler, C.; Wiesner, M. R.; Lowry, G. V., Speciation matters: Bioavailability of silver and silver sulfide nanoparticles to alfalfa (Medicago sativa). *Environmental science & technology* **2015,** 49, (14), 8451-8460.

50. Ma, C.; Chhikara, S.; Minocha, R.; Long, S.; Musante, C.; White, J. C.; Xing, B.; Dhankher, O. P., Reduced silver nanoparticle phytotoxicity in Crambe abyssinica with enhanced glutathione production by overexpressing bacterial γ-glutamylcysteine synthase. *Environmental science & technology* **2015,** 49, (16), 10117-10126.
51. Yin, L.; Cheng, Y.; Espinasse, B.; Colman, B. P.; Auffan, M.; Wiesner, M.; Rose, J.; Liu, J.; Bernhardt, E. S., More than the ions: the effects of silver nanoparticles on Lolium multiflorum. *Environmental science & technology* **2011,** 45, (6), 2360-2367.
52. Kaveh, R.; Li, Y.-S.; Ranjbar, S.; Tehrani, R.; Brueck, C. L.; Van Aken, B., Changes in Arabidopsis thaliana gene expression in response to silver nanoparticles and silver ions. *Environmental science & technology* **2013,** 47, (18), 10637-10644.
53. Wang, J.; Koo, Y.; Alexander, A.; Yang, Y.; Westerhof, S.; Zhang, Q.; Schnoor, J. L.; Colvin, V. L.; Braam, J.; Alvarez, P. J., Phytostimulation of poplars and Arabidopsis exposed to silver nanoparticles and Ag+ at sublethal concentrations. *Environmental science & technology* **2013,** 47, (10), 5442-5449.
54. Geisler-Lee, J.; Wang, Q.; Yao, Y.; Zhang, W.; Geisler, M.; Li, K.; Huang, Y.; Chen, Y.; Kolmakov, A.; Ma, X., Phytotoxicity, accumulation and transport of silver nanoparticles by Arabidopsis thaliana. *Nanotoxicology* **2012,** 7, (3), 323-337.
55. Song, U.; Jun, H.; Waldman, B.; Roh, J.; Kim, Y.; Yi, J.; Lee, E. J., Functional analyses of nanoparticle toxicity: a comparative study of the effects of TiO2 and Ag on tomatoes (Lycopersicon esculentum). *Ecotoxicology and environmental safety* **2013,** 93, 60-67.
56. Coleman, J. G.; Kennedy, A. J.; Bednar, A. J.; Ranville, J. F.; Laird, J. G.; Harmon, A. R.; Hayes, C. A.; Gray, E. P.; Higgins, C. P.; Lotufo, G., Comparing the effects of nanosilver size and coating variations on bioavailability, internalization, and elimination, using Lumbriculus variegatus. *Environmental toxicology and chemistry* **2013,** 32, (9), 2069-2077.
57. Li, C.-C.; Dang, F.; Li, M.; Zhu, M.; Zhong, H.; Hintelmann, H.; Zhou, D.-M., Effects of exposure pathways on the accumulation and phytotoxicity of silver nanoparticles in soybean and rice. *Nanotoxicology* **2017,** 11, (5), 699-709.
58. Bao, D.; Oh, Z. G.; Chen, Z., Characterization of silver nanoparticles internalized by Arabidopsis plants using single particle ICP-MS analysis. *Frontiers in plant science* **2016,** 7, 32.
59. Zhao, C.-M.; Wang, W.-X., Importance of surface coatings and soluble silver in silver nanoparticles toxicity to Daphnia magna. *Nanotoxicology* **2012,** 6, (4), 361-370.
60. Zhao, C.-M.; Wang, W.-X., Size-dependent uptake of silver nanoparticles in Daphnia magna. *Environmental science & technology* **2012,** 46, (20), 11345-11351.
61. Asztemborska, M.; Jakubiak, M.; Książyk, M.; Stęborowski, R.; Polkowska-Motrenko, H.; Bystrzejewska-Piotrowska, G., Silver nanoparticle accumulation by aquatic organisms–neutron activation as a tool for the environmental fate of nanoparticles tracing. *Nukleonika* **2014,** 59, (4), 169-173.
62. Al-Sid-Cheikh, M.; Rouleau, C.; Pelletier, E., Tissue distribution and kinetics of dissolved and nanoparticulate silver in Iceland scallop (Chlamys islandica). *Marine environmental research* **2013,** 86, 21-28.
63. Coutris, C.; Hertel-Aas, T.; Lapied, E.; Joner, E. J.; Oughton, D. H., Bioavailability of cobalt and silver nanoparticles to the earthworm Eisenia fetida. *Nanotoxicology* **2012,** 6, (2), 186-195.
64. Jung, Y.-J.; Kim, K.-T.; Kim, J. Y.; Yang, S.-Y.; Lee, B.-G.; Kim, S. D., Bioconcentration and distribution of silver nanoparticles in Japanese medaka (Oryzias latipes). *Journal of hazardous materials* **2014,** 267, 206-213.
65. Ishwarya, R.; Vaseeharan, B.; Shanthi, S.; Ramesh, S.; Manogari, P.; Dhanalakshmi, K.; Vijayakumar, S.; Benelli, G., Green synthesized silver nanoparticles: toxicity against Poecilia reticulata fishes and Ceriodaphnia cornuta crustaceans. *Journal of Cluster Science* **2017,** 28, (1), 519-527.
66. Yoo-Iam, M.; Chaichana, R.; Satapanajaru, T., Toxicity, bioaccumulation and biomagnification of silver nanoparticles in green algae (Chlorella sp.), water flea (Moina macrocopa), blood worm

(Chironomus spp.) and silver barb (Barbonymus gonionotus). *Chemical Speciation & Bioavailability* **2014,** 26, (4), 257-265.
67. Mortimer, M.; Gogos, A.; Bartolomé, N.; Kahru, A.; Bucheli, T. D.; Slaveykova, V. I., Potential of hyperspectral imaging microscopy for semi-quantitative analysis of nanoparticle uptake by protozoa. *Environmental science & technology* **2014,** 48, (15), 8760-8767.
68. Yang, X.; Jiang, C.; Hsu-Kim, H.; Badireddy, A. R.; Dykstra, M.; Wiesner, M.; Hinton, D. E.; Meyer, J. N., Silver nanoparticle behavior, uptake, and toxicity in Caenorhabditis elegans: effects of natural organic matter. *Environmental science & technology* **2014,** 48, (6), 3486-3495.
69. Kwok, K. W.; Auffan, M.; Badireddy, A. R.; Nelson, C. M.; Wiesner, M. R.; Chilkoti, A.; Liu, J.; Marinakos, S. M.; Hinton, D. E., Uptake of silver nanoparticles and toxicity to early life stages of Japanese medaka (Oryzias latipes): effect of coating materials. *Aquatic toxicology* **2012,** 120, 59-66.
70. Tkalec, Ž. P.; Drobne, D.; Vogel-Mikuš, K.; Pongrac, P.; Regvar, M.; Štrus, J.; Pelicon, P.; Vavpetič, P.; Grlj, N.; Remškar, M., Micro-PIXE study of Ag in digestive glands of a nano-Ag fed arthropod (Porcellio scaber, Isopoda, Crustacea). *Nuclear Instruments and Methods in Physics Research Section B: Beam Interactions with Materials and Atoms* **2011,** 269, (20), 2286-2291.
71. García-Alonso, J.; Khan, F. R.; Misra, S. K.; Turmaine, M.; Smith, B. D.; Rainbow, P. S.; Luoma, S. N.; Valsami-Jones, E., Cellular internalization of silver nanoparticles in gut epithelia of the estuarine polychaete Nereis diversicolor. *Environmental science & technology* **2011,** 45, (10), 4630-4636.
72. Lee, K. J.; Browning, L. M.; Nallathamby, P. D.; Desai, T.; Cherukuri, P. K.; Xu, X.-H. N., In vivo quantitative study of sized-dependent transport and toxicity of single silver nanoparticles using zebrafish embryos. *Chemical research in toxicology* **2012,** 25, (5), 1029-1046.
73. Sekine, R.; Moore, K. L.; Matzke, M.; Vallotton, P.; Jiang, H.; Hughes, G. M.; Kirby, J. K.; Donner, E.; Grovenor, C. R.; Svendsen, C., Complementary imaging of silver nanoparticle interactions with green algae: dark-field microscopy, electron microscopy, and nanoscale secondary ion mass spectrometry. *ACS nano* **2017,** 11, (11), 10894-10902.
74. Larue, C.; Castillo-Michel, H.; Sobanska, S.; Cécillon, L.; Bureau, S.; Barthès, V.; Ouerdane, L.; Carrière, M.; Sarret, G., Foliar exposure of the crop Lactuca sativa to silver nanoparticles: evidence for internalization and changes in Ag speciation. *Journal of hazardous materials* **2014,** 264, 98-106.
75. Auffan, M.; Matson, C. W.; Rose, J.; Arnold, M.; Proux, O.; Fayard, B.; Liu, W.; Chaurand, P.; Wiesner, M. R.; Bottero, J.-Y., Salinity-dependent silver nanoparticle uptake and transformation by Atlantic killifish (Fundulus heteroclitus) embryos. *Nanotoxicology* **2014,** 8, (sup1), 167-176.
76. Starnes, D. L.; Unrine, J. M.; Starnes, C. P.; Collin, B. E.; Oostveen, E. K.; Ma, R.; Lowry, G. V.; Bertsch, P. M.; Tsyusko, O. V., Impact of sulfidation on the bioavailability and toxicity of silver nanoparticles to Caenorhabditis elegans. *Environmental pollution* **2015,** 196, 239-246.
77. Wang, H.; Ho, K. T.; Scheckel, K. G.; Wu, F.; Cantwell, M. G.; Katz, D. R.; Horowitz, D. B.; Boothman, W. S.; Burgess, R. M., Toxicity, bioaccumulation, and biotransformation of silver nanoparticles in marine organisms. *Environmental science & technology* **2014,** 48, (23), 13711-13717.
78. Stegemeier, J. P.; Colman, B. P.; Schwab, F.; Wiesner, M. R.; Lowry, G. V., Uptake and Distribution of Silver in the Aquatic Plant Landoltia punctata (Duckweed) Exposed to Silver and Silver Sulfide Nanoparticles. *Environmental Science & Technology* **2017,** 51, (9), 4936-4943.
79. Roh, J.-y.; Sim, S. J.; Yi, J.; Park, K.; Chung, K. H.; Ryu, D.-y.; Choi, J., Ecotoxicity of silver nanoparticles on the soil nematode Caenorhabditis elegans using functional ecotoxicogenomics. *Environmental science & technology* **2009,** 43, (10), 3933-3940.
80. Cambier, S.; Røgeberg, M.; Georgantzopoulou, A.; Serchi, T.; Karlsson, C.; Verhaegen, S.; Iversen, T.-G.; Guignard, C.; Kruszewski, M.; Hoffmann, L., Fate and effects of silver nanoparticles on early life-stage development of zebrafish (Danio rerio) in comparison to silver nitrate. *Science of The Total Environment* **2018,** 610, 972-982.

81. Böhme, S.; Baccaro, M.; Schmidt, M.; Potthoff, A.; Stärk, H.-J.; Reemtsma, T.; Kühnel, D., Metal uptake and distribution in the zebrafish (Danio rerio) embryo: differences between nanoparticles and metal ions. *Environmental Science: Nano* **2017,** 4, (5), 1005-1015.
82. Lee, K. J.; Nallathamby, P. D.; Browning, L. M.; Osgood, C. J.; Xu, X.-H. N., In vivo imaging of transport and biocompatibility of single silver nanoparticles in early development of zebrafish embryos. *ACS nano* **2007,** 1, (2), 133-143.
83. Wang, S.; Lv, J.; Ma, J.; Zhang, S., Cellular internalization and intracellular biotransformation of silver nanoparticles in Chlamydomonas reinhardtii. *Nanotoxicology* **2016,** 10, (8), 1129-1135.
84. McGillicuddy, E.; Murray, I.; Kavanagh, S.; Morrison, L.; Fogarty, A.; Cormican, M.; Dockery, P.; Prendergast, M.; Rowan, N.; Morris, D., Silver nanoparticles in the environment: Sources, detection and ecotoxicology. *Science of the Total Environment* **2017,** 575, 231-246.
85. Schultz, C.; Powell, K.; Crossley, A.; Jurkschat, K.; Kille, P.; Morgan, A. J.; Read, D.; Tyne, W.; Lahive, E.; Svendsen, C., Analytical approaches to support current understanding of exposure, uptake and distributions of engineered nanoparticles by aquatic and terrestrial organisms. *Ecotoxicology* **2015,** 24, (2), 239-261.
86. Bandyopadhyay, S.; Peralta-Videa, J. R.; Gardea-Torresdey, J. L., Advanced analytical techniques for the measurement of nanomaterials in food and agricultural samples: a review. *Environmental engineering science* **2013,** 30, (3), 118-125.
87. Bandyopadhyay, S.; Peralta-Videa, J. R.; Hernandez-Viezcas, J. A.; Montes, M. O.; Keller, A. A.; Gardea-Torresdey, J. L., Microscopic and spectroscopic methods applied to the measurements of nanoparticles in the environment. *Applied Spectroscopy Reviews* **2012,** 47, (3), 180-206.
88. Tyler, G.; Jobin Yvon, S., ICP-OES, ICP-MS and AAS Techniques Compared. *ICP Optical Emission Spectroscopy Technical Note* **1995,** 5.
89. Silvestre, M.; Lagarda, M.; Farré, R.; Martınez-Costa, C.; Brines, J., Copper, iron and zinc determinations in human milk using FAAS with microwave digestion. *Food Chemistry* **2000,** 68, (1), 95-99.
90. Benn, T. M.; Westerhoff, P., Nanoparticle silver released into water from commercially available sock fabrics. *Environmental science & technology* **2008,** 42, (11), 4133-4139.
91. Laborda, F.; Bolea, E.; Jiménez-Lamana, J., Single particle inductively coupled plasma mass spectrometry: a powerful tool for nanoanalysis. ACS Publications: 2013.
92. Mitrano, D. M.; Barber, A.; Bednar, A.; Westerhoff, P.; Higgins, C. P.; Ranville, J. F., Silver nanoparticle characterization using single particle ICP-MS (SP-ICP-MS) and asymmetrical flow field flow fractionation ICP-MS (AF4-ICP-MS). *Journal of Analytical Atomic Spectrometry* **2012,** 27, (7), 1131-1142.
93. Loeschner, K.; Navratilova, J.; Købler, C.; Mølhave, K.; Wagner, S.; von der Kammer, F.; Larsen, E. H., Detection and characterization of silver nanoparticles in chicken meat by asymmetric flow field flow fractionation with detection by conventional or single particle ICP-MS. *Analytical and bioanalytical chemistry* **2013,** 405, (25), 8185-8195.
94. Melnik, E.; Demin, V.; Demin, V.; Gmoshinski, I.; Tyshko, N.; Tutelyan, V., Transfer of silver nanoparticles through the placenta and breast milk during in vivo experiments on rats. *Acta Naturae (англоязычная версия)* **2013,** 5, (3 (18)).
95. Larner, F.; Dogra, Y.; Dybowska, A.; Fabrega, J.; Stolpe, B. r.; Bridgestock, L. J.; Goodhead, R.; Weiss, D. J.; Moger, J.; Lead, J. R., Tracing bioavailability of ZnO nanoparticles using stable isotope labeling. *Environmental science & technology* **2012,** 46, (21), 12137-12145.
96. Yin, Y.; Tan, Z.; Hu, L.; Yu, S.; Liu, J.; Jiang, G., Isotope tracers to study the environmental fate and bioaccumulation of metal-containing engineered nanoparticles: techniques and applications. *Chem. Rev* **2017,** 117, (5), 4462-4487.
97. Zuykov, M.; Pelletier, E.; Demers, S., Colloidal complexed silver and silver nanoparticles in extrapallial fluid of Mytilus edulis. *Marine environmental research* **2011,** 71, (1), 17-21.
98. Coutris, C.; Joner, E. J.; Oughton, D. H., Aging and soil organic matter content affect the fate of silver nanoparticles in soil. *Science of the Total Environment* **2012,** 420, 327-333.

99. Günther, D.; Hattendorf, B., Solid sample analysis using laser ablation inductively coupled plasma mass spectrometry. *TrAC Trends in Analytical Chemistry* **2005,** 24, (3), 255-265.
100. Pozebon, D.; Scheffler, G.; Dressler, V., Recent applications of laser ablation inductively coupled plasma mass spectrometry (LA-ICP-MS) for biological sample analysis: a follow-up review. *Journal of Analytical Atomic Spectrometry* **2017,** 32, (5), 890-919.
101. Hare, D. J.; Kysenius, K.; Paul, B.; Knauer, B.; Hutchinson, R. W.; O'Connor, C.; Fryer, F.; Hennessey, T. P.; Bush, A. I.; Crouch, P. J., Imaging Metals in Brain Tissue by Laser Ablation-Inductively Coupled Plasma-Mass Spectrometry (LA-ICP-MS). *Journal of visualized experiments: JoVE* **2017,** (119).
102. Sodhi, R. N., Time-of-flight secondary ion mass spectrometry (TOF-SIMS):—versatility in chemical and imaging surface analysis. *Analyst* **2004,** 129, (6), 483-487.
103. Senoner, M.; Unger, W. E., SIMS imaging of the nanoworld: applications in science and technology. *Journal of Analytical Atomic Spectrometry* **2012,** 27, (7), 1050-1068.
104. Benninghoven, A., Chemical analysis of inorganic and organic surfaces and thin films by static time-of-flight secondary ion mass spectrometry (TOF-SIMS). *Angewandte Chemie International Edition in English* **1994,** 33, (10), 1023-1043.
105. Herrmann, A. M.; Ritz, K.; Nunan, N.; Clode, P. L.; Pett-Ridge, J.; Kilburn, M. R.; Murphy, D. V.; O'Donnell, A. G.; Stockdale, E. A., Nano-scale secondary ion mass spectrometry—a new analytical tool in biogeochemistry and soil ecology: a review article. *Soil Biology and Biochemistry* **2007,** 39, (8), 1835-1850.
106. Hagenhoff, B., High resolution surface analysis by TOF-SIMS. *Microchimica Acta* **2000,** 132, (2-4), 259-271.
107. Hu, M.; Novo, C.; Funston, A.; Wang, H.; Staleva, H.; Zou, S.; Mulvaney, P.; Xia, Y.; Hartland, G. V., Dark-field microscopy studies of single metal nanoparticles: understanding the factors that influence the linewidth of the localized surface plasmon resonance. *Journal of materials chemistry* **2008,** 18, (17), 1949-1960.
108. Browning, L. M.; Huang, T.; Xu, X.-H. N., Real-time in vivo imaging of size-dependent transport and toxicity of gold nanoparticles in zebrafish embryos using single nanoparticle plasmonic spectroscopy. *Interface Focus* **2013,** 3, (3), 20120098.
109. Lindfors, K.; Kalkbrenner, T.; Stoller, P.; Sandoghdar, V., Detection and spectroscopy of gold nanoparticles using supercontinuum white light confocal microscopy. *Physical review letters* **2004,** 93, (3), 037401.
110. Beach, J., Hyperspectral imaging. *BioOpt World* **2009,** 3, 2-4.
111. Badireddy, A. R.; Wiesner, M. R.; Liu, J., Detection, characterization, and abundance of engineered nanoparticles in complex waters by hyperspectral imagery with enhanced darkfield microscopy. *Environmental science & technology* **2012,** 46, (18), 10081-10088.
112. Yang, X.; Gondikas, A. P.; Marinakos, S. M.; Auffan, M.; Liu, J.; Hsu-Kim, H.; Meyer, J. N., Mechanism of silver nanoparticle toxicity is dependent on dissolved silver and surface coating in Caenorhabditis elegans. *Environmental science & technology* **2011,** 46, (2), 1119-1127.
113. Meyer, J. N.; Lord, C. A.; Yang, X. Y.; Turner, E. A.; Badireddy, A. R.; Marinakos, S. M.; Chilkoti, A.; Wiesner, M. R.; Auffan, M., Intracellular uptake and associated toxicity of silver nanoparticles in Caenorhabditis elegans. *Aquatic toxicology* **2010,** 100, (2), 140-150.
114. Kaulich, B.; Gianoncelli, A.; Beran, A.; Eichert, D.; Kreft, I.; Pongrac, P.; Regvar, M.; Vogel-Mikuš, K.; Kiskinova, M., Low-energy X-ray fluorescence microscopy opening new opportunities for bio-related research. *Journal of the Royal Society interface* **2009,** 6, (Suppl 5), S641-S647.
115. Utsunomiya, S.; Ewing, R. C., Application of high-angle annular dark field scanning transmission electron microscopy, scanning transmission electron microscopy-energy dispersive X-ray spectrometry, and energy-filtered transmission electron microscopy to the characterization of nanoparticles in the environment. *Environmental Science & Technology* **2003,** 37, (4), 786-791.
116. Kim, S. W.; Nam, S.-H.; An, Y.-J., Interaction of silver nanoparticles with biological surfaces of Caenorhabditis elegans. *Ecotoxicology and environmental safety* **2012,** 77, 64-70.

117. Luo, X.; Xu, S.; Yang, Y.; Li, L.; Chen, S.; Xu, A.; Wu, L., Insights into the ecotoxicity of silver nanoparticles transferred from Escherichia coli to Caenorhabditis elegans. *Scientific reports* **2016,** 6, 36465.
118. Drobne, D., 3D imaging of cells and tissues by focused ion beam/scanning electron microscopy (FIB/SEM). In *Nanoimaging*, Springer: 2013; pp 275-292.
119. Drobne, D.; Milani, M.; Zrimec, A.; Lešer, V.; Berden Zrimec, M., Electron and ion imaging of gland cells using the FIB/SEM system. *Journal of microscopy* **2005,** 219, (1), 29-35.
120. Guehrs, E.; Schneider, M.; Günther, C. M.; Hessing, P.; Heitz, K.; Wittke, D.; Oliver, A. L.-S.; Jakubowski, N.; Plendl, J.; Eisebitt, S., Quantification of silver nanoparticle uptake and distribution within individual human macrophages by FIB/SEM slice and view. *Journal of nanobiotechnology* **2017,** 15, (1), 21.
121. Johansson, S. A.; Campbell, J. L.; Malmqvist, K. G., *Particle-induced X-ray emission spectrometry (PIXE)*. John Wiley & Sons: 1995; Vol. 133.
122. Vogel-Mikuš, K.; Pelicon, P.; Vavpetič, P.; Kreft, I.; Regvar, M., Elemental analysis of edible grains by micro-PIXE: common buckwheat case study. *Nuclear Instruments and Methods in Physics Research Section B: Beam Interactions with Materials and Atoms* **2009,** 267, (17), 2884-2889.
123. Trushin, M.; Seifert, W.; Vyvenko, O.; Bauer, J.; Martinez-Criado, G.; Salome, M.; Kittler, M., XBIC/μ-XRF/μ-XAS analysis of metals precipitation in block-cast solar silicon. *Nuclear Instruments and Methods in Physics Research Section B: Beam Interactions with Materials and Atoms* **2010,** 268, (3-4), 254-258.
124. Kawai, J., Absorption Techniques in X-Ray Spectrometry. *Encyclopedia of Analytical Chemistry* **2000**.
125. George, G. N.; Pickering, I. J.; Pushie, M. J.; Nienaber, K.; Hackett, M. J.; Ascone, I.; Hedman, B.; Hodgson, K. O.; Aitken, J. B.; Levina, A., X-ray-induced photo-chemistry and X-ray absorption spectroscopy of biological samples. *Journal of synchrotron radiation* **2012,** 19, (6), 875-886.
126. Lombi, E.; Hettiarachchi, G. M.; Scheckel, K. G., Advanced in situ spectroscopic techniques and their applications in environmental biogeochemistry: Introduction to the special section. *Journal of environmental quality* **2011,** 40, (3), 659-666.
127. Betz, O.; Wegst, U.; Weide, D.; Heethoff, M.; Helfen, L.; LEE, W. K.; Cloetens, P., Imaging applications of synchrotron X-ray phase-contrast microtomography in biological morphology and biomaterials science. I. General aspects of the technique and its advantages in the analysis of millimetre-sized arthropod structure. *Journal of Microscopy* **2007,** 227, (1), 51-71.
128. Eichert, D.; Gregoratti, L.; Kaulich, B.; Marcello, A.; Melpignano, P.; Quaroni, L.; Kiskinova, M., Imaging with spectroscopic micro-analysis using synchrotron radiation. *Analytical and bioanalytical chemistry* **2007,** 389, (4), 1121-1132.
129. Yano, J.; Yachandra, V. K., X-ray absorption spectroscopy. *Photosynthesis research* **2009,** 102, (2-3), 241.
